# Suppression of human and simian immunodeficiency virus replication with the CCR5-specific antibody Leronlimab in two species

**DOI:** 10.1371/journal.ppat.1010396

**Published:** 2022-03-31

**Authors:** Xiao L. Chang, Jason S. Reed, Gabriela M. Webb, Helen L. Wu, Jimmy Le, Katherine B. Bateman, Justin M. Greene, Cleiton Pessoa, Courtney Waytashek, Whitney C. Weber, Joseph Hwang, Miranda Fischer, Cassandra Moats, Oriene Shiel, Rachele M. Bochart, Hugh Crank, Don Siess, Travis Giobbi, Jeffrey Torgerson, Rebecca Agnor, Lina Gao, Kush Dhody, Jacob P. Lalezari, Ivo Sah Bandar, Alnor M. Carnate, Alina S. Pang, Michael J. Corley, Scott Kelly, Nader Pourhassan, Jeremy Smedley, Benjamin N. Bimber, Scott G. Hansen, Lishomwa C. Ndhlovu, Jonah B. Sacha

**Affiliations:** 1 Vaccine & Gene Therapy Institute, Oregon Health & Science University, Portland, Oregon, United States of America; 2 Oregon National Primate Research Center, Oregon Health & Science University, Portland, Oregon, United States of America; 3 Quest Clinical Research, San Francisco, California, United States of America; 4 Amarex Clinical Research LLC, Germantown, Maryland, United States of America; 5 Department of Medicine, Division of Infectious Diseases, Weill Cornell Medicine, New York, New York, United States of America; 6 CytoDyn Inc., Vancouver, Washington, United States of America; University of North Carolina at Chapel Hill, UNITED STATES

## Abstract

The CCR5-specific antibody Leronlimab is being investigated as a novel immunotherapy that can suppress HIV replication with minimal side effects. Here we studied the virological and immunological consequences of Leronlimab in chronically CCR5-tropic HIV-1 infected humans (n = 5) on suppressive antiretroviral therapy (ART) and in ART-naïve acutely CCR5-tropic SHIV infected rhesus macaques (n = 4). All five human participants transitioned from daily combination ART to self-administered weekly subcutaneous (SC) injections of 350 mg or 700 mg Leronlimab and to date all participants have sustained virologic suppression for over seven years. In all participants, Leronlimab fully occupied CCR5 receptors on peripheral blood CD4+ T cells and monocytes. In ART-naïve rhesus macaques acutely infected with CCR5-tropic SHIV, weekly SC injections of 50 mg/kg Leronlimab fully suppressed plasma viremia in half of the macaques. CCR5 receptor occupancy by Leronlimab occurred concomitant with rebound of CD4+ CCR5+ T-cells in peripheral blood, and full CCR5 receptor occupancy was found in multiple anatomical compartments. Our results demonstrate that weekly, self-administered Leronlimab was safe, well-tolerated, and efficacious for long-term virologic suppression and should be included in the arsenal of safe, easily administered, longer-acting antiretroviral treatments for people living with HIV-1.

**Trial Registration:** ClinicalTrials.gov Identifiers: NCT02175680 and NCT02355184.

## Author summary

With approximately 37 million people living with HIV, stopping the HIV epidemic remains a top global health priority. While daily oral antiretroviral therapy limits HIV replication, its use is a lifelong requirement and increases the likelihood for the development of drug-resistant variants. Indeed, the global prevalence of HIV drug resistance has exponentially increased in recent years, leading to a need for new drug targets. CCR5 is an ideal drug target as HIV uses this molecule to gain entry into target cells. As an example of the importance of CCR5, individuals that lack CCR5 expression due to a natural genetic mutation are naturally resistant to HIV infection. Here, we report that weekly injections of Leronlimab, an anti-CCR5 antibody that blocks the binding of HIV to CCR5, suppressed HIV replication in five HIV+ participants for over seven years. When used to treat acutely infected rhesus macaques, we found that the average amount of virus in the blood of Leronlimab-treated macaques was 10,000 times lower than in untreated macaques. These data suggest that Leronlimab is a safe and effective anti-HIV therapeutic drug.

## Introduction

Combination antiretroviral therapy (ART) has drastically improved survival and increased life expectancy for people living with HIV [1]. However, it is not curative and requires daily adherence for life. Furthermore, the prevalence of antiretroviral (ARV) drug resistance has significantly increased in recent years [2], with future projections for increasing AIDS-related deaths, new infections, and costs for HIV maintenance all attributable to ART failure due to drug resistance [3]. To address this problem, the World Health Organization emphasized the need for new ART strategies in their Global Action Plan on HIV Drug Resistance [4].

Both small-molecule ARVs [5] and broadly neutralizing antibodies (bNAbs) [6] can lead to the emergence of drug-resistant variants. Alternatively, a promising approach may be to target the conserved major coreceptor CCR5 used by HIV-1 for CD4+ T-cell entry. Indeed, individuals homozygous for a 32-base pair deletion in *ccr5* are highly resistant to infection by HIV-1 strains encoding a variety of CCR5-tropic envelope sequences [7]. Furthermore, recent attempts in curative strategies have been directed at CCR5 [8], making it an ideal target for HIV-1 therapies.

Leronlimab (PRO 140, Vyrologix) is an anti-CCR5 humanized monoclonal IgG4 antibody tested in multiple clinical trials for HIV therapy as a weekly SC injection, with favorable safety and tolerability profiles in over 1,000 volunteers [9]. Similar to HIV, Leronlimab binds to the extracellular loop-2 and N-terminus domains of CCR5, thereby directly outcompeting HIV for CCR5 engagement and blocking entry to CD4+ T-cells [10]. This mechanism of action is in contrast to the FDA-approved CCR5 small-molecule inhibitor maraviroc that binds to CCR5 by allosteric modulation, allowing maraviroc-resistant HIV variants to utilize maraviroc-bound CCR5 for cell entry [11,12]. In contrast, viral susceptibility to Leronlimab did not change after Leronlimab treatment [13,14], demonstrating its high genetic barrier to viral resistance. Additionally, a single Leronlimab injection reduced plasma viremia by approximately 100-fold [13,15] while, in the CD01 study (ClinicalTrials.gov NCT02175680), weekly SC Leronlimab maintained virologic suppression in 56.1% of CCR5-tropic HIV-infected participants (n = 41) for 12 consecutive weeks after ART interruption [14]. Here, we report that uninterrupted weekly Leronlimab treatments resulted in over seven years of virologic suppression in five chronically CCR5-tropic HIV-infected human participants and reduced plasma viremia by 4.29 log_10_ in four acutely CCR5-tropic SHIV-infected rhesus macaques, demonstrating long-term safety and antiviral efficacy of Leronlimab for HIV-1 therapies.

## Results

### Leronlimab treatment in HIV-infected human participants for over seven years

**Fig 1:**
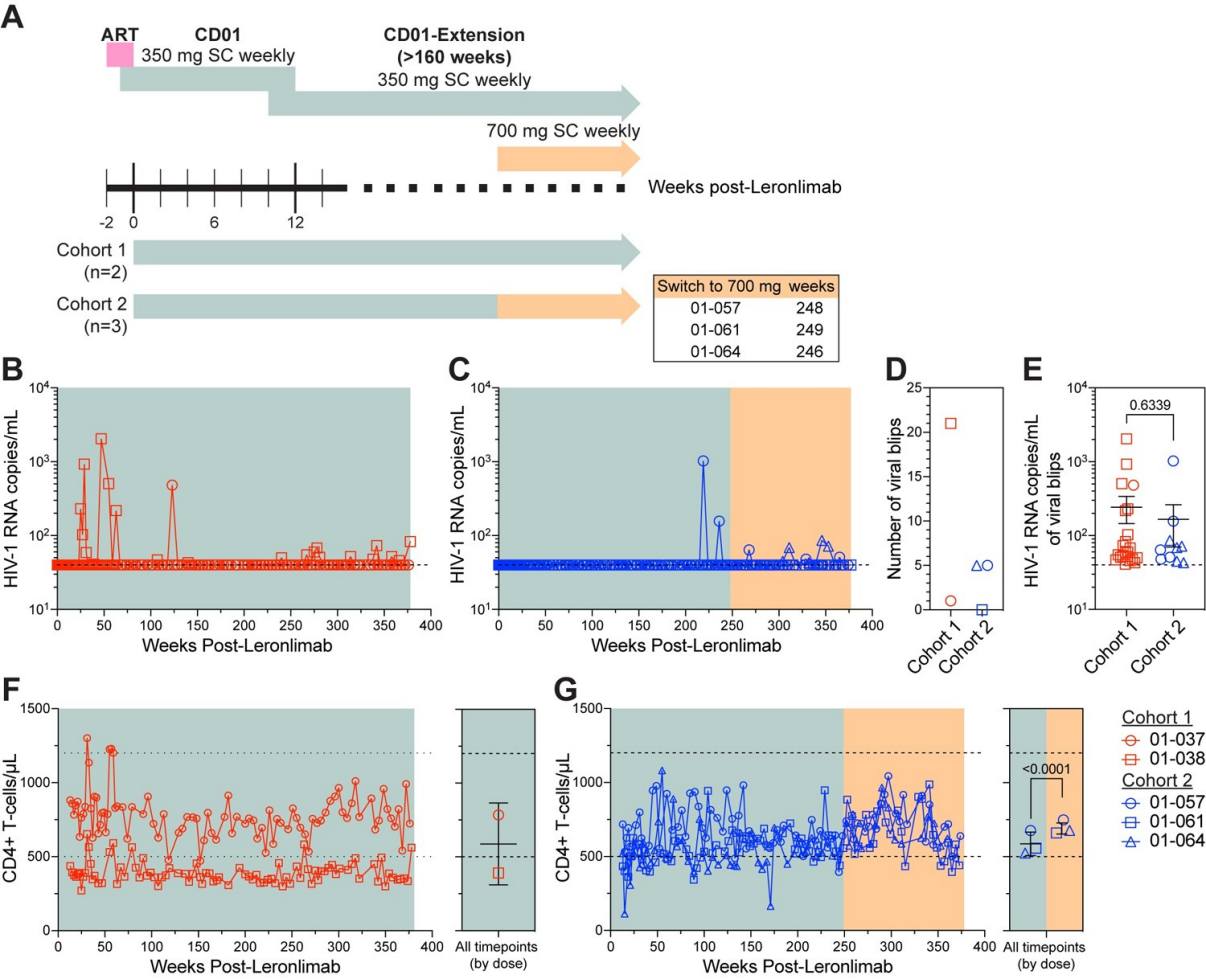
Leronlimab maintained virologic suppression in humans for over seven years. **(A)** Study outline for CD01 and CD01-Extension studies (ClinicalTrials.gov: NCT02175680 and NCT02355184). One-week prior to ART interruption, cohort 1 (n = 2; red) and cohort 2 (n = 3; blue) received weekly SC 350 mg injections. After weeks 246–249, cohort 2 switched to weekly SC 700 mg. Longitudinal HIV-1 RNA copies/mL in plasma for **(B)** cohort 1 and **(C)** cohort 2. **(D)** Total number and **(E)** mean (±SEM) HIV-1 RNA copies/mL of viral blips (>40 copies/mL). (B-C, E) Horizontal dashed line denotes assay limit of detection (LOD) of 40 copies/mL; undetected viral loads graphed at LOD. **(F-G)** Left graph shows longitudinal CD4+ T-cell counts in the blood and right graph shows mean (±SD) for all CD4+ T-cell timepoints based on the treatment dose for cohort 1 (E) and cohort 2 (F). (F-G) Two horizontal dotted lines represent the normal range for CD4+ T-cells. Gray and orange boxes denote 350 and 700 mg injections, respectively. (E, G) Two-tailed unpair t test.

To evaluate the long-term safety, tolerability, and efficacy of weekly SC Leronlimab in humans, ten virologically-suppressed participants from the CD01 study (ClinicalTrials.gov NCT02175680) were enrolled in the CD01-Extension study (ClinicalTrials.gov NCT02355184) to continue self-administration of weekly Leronlimab for at least 160 weeks. Of these ten, four individuals experienced viral rebound and stopped monotherapy, and one individual withdrew, leaving five long-term participants (Fig 1A and Table 1). In this ongoing study, cohort 1 (n = 2) received weekly 350 mg Leronlimab and cohort 2 (n = 3) increased their weekly doses from 350 mg to 700 mg. As previously reported, Leronlimab was well-tolerated [14] and no sustained changes were observed in longitudinal serum chemistry profiles (S1 Fig).

**Table 1:** Human participant demographics and clinical information.

ID	Sex	Age (Years)	Average weight (kg)	Duration of HIV infection (years)	Prior ART regimen	Class	CCR5 Δ32	CCR5 haplotype
01–037	Male	39.9	70	11	TDF/FTC/RPV	NRTI/NNRTI	WT/WT	HHA/HHE
01–038	Male	63.7	80.5	38	RAL/DTG	INSTI	WT/Δ32	HHE/HHG*2
01–057	Male	32.6	83.5	16	TDF/FTC/EVG/c	INSTI/ NRTI	WT/WT	HHD/HHG*1
01–061	Female	73.8	59.5	10	TDF/FTC	NRTI	WT/WT	HHA/HHF
01–064	Male	51.9	97	10	TDF/FTC/EVG/c	INSTI/ NRTI	WT/WT	HHC/HHE

TDF = tenofovir disoproxil fumarate; FTC = emtricitabine; RPV = rilpivirine; RAL = raltegravir; DTG = dolutegravir; EVG/c = elvitegravir/cobicistat; NRTI = nucleoside reverse transcriptase inhibitor; NNRTI = non-nucleoside reverse transcriptase inhibitor; INSTI = integrase strand transfer inhibitor. WT = Wild type

Leronlimab treatment maintained virologic suppression in all five participants for over seven years (Fig 1B and 1C). Four of the five participants experienced infrequent, transient episodes of plasma viremia, termed viral blips, while one participant from cohort 2 (01–061) was aviremic in all sampled timepoints (Fig 1D and S1 Table). Participant 01–038, who has the longest duration HIV infection of all study participants at 38 years, presented with the highest number of blips. Viral blips occurred in 11.6% of the sampled timepoints (n = 189) in cohort 1 and 3.8% of the sampled timepoints (n = 262) in cohort 2. Although the viral blip frequency for all participants overall at 7.1% was higher than the 2.0% observed in 4,449 sampled timepoints from 735 HIV-infected individuals on long-term ART [16], the small sample size precludes statistical analysis. Mean (± SEM) plasma viral loads during these transient viremic episodes were 242.4 (± 456.3) and 166.0 (± 303.7) HIV-1 copies/mL for cohort 1 and 2, respectively (Fig 1E). Lastly, viral blips in most participants spontaneously resolved by the next timepoint, indicating lack of emergence of Leronlimab-resistant variants.

Low CD4+ T-cell counts in the blood is a hallmark of untreated HIV. Here, four of the five participants had longitudinal CD4+ T-cell counts within the normal reference range (Fig 1F and 1G), showing that long-term Leronlimab use does not negatively impact CD4+ T cell levels. In fact, the dose change from 350 to 700 mg sequentially increased CD4+ T-cell counts in all three participants from cohort 2 (p<0.0001). Notably, 01–038 was the only participant with CD4+ T-cell counts below the normal range, likely attributable to the length of HIV infection in this participant.

**Fig 2:**
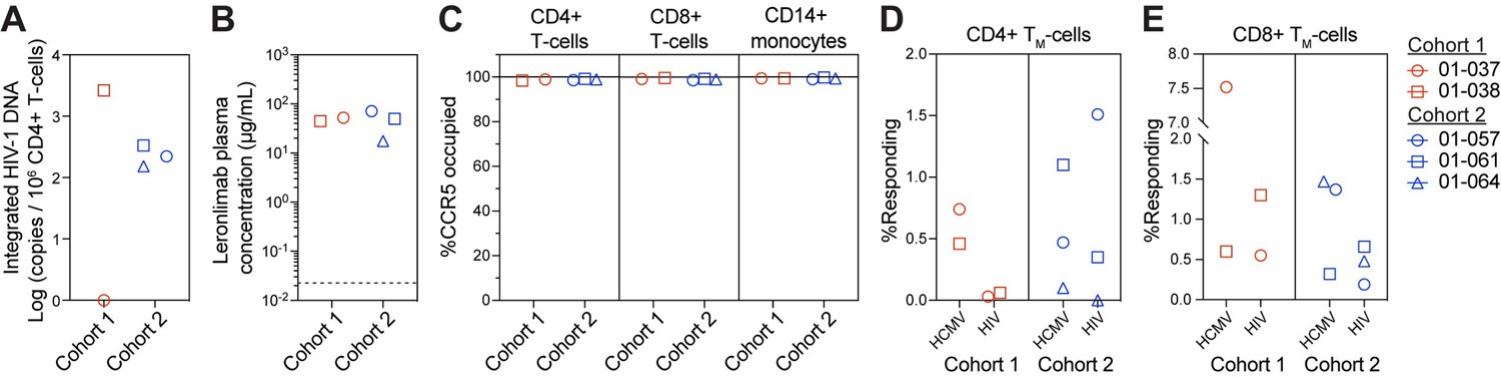
Analyses of long-term Leronlimab treatment in humans. Blood was collected from 01–037, 01–038, 01–057, 01–061, and 01–064 at weeks 304, 301, 297, 299, and 297, respectively. **(A)** Integrated HIV-1 DNA copies in CD4+ T-cells. **(B)** Leronlimab concentration in the plasma. Horizontal dashed line denotes LOD (0.0226 μg/mL). **(C)** CCR5 RO by Leronlimab on CD4+ T-cells, CD8+ T-cells, and CD14+ monocytes. Intracellular cytokine staining of CD95+ memory **(D)** CD4+ and **(E)** CD8+ T_M_-cells stimulated with HCMV (IE1 and PP65) and HIV (Gag and Nef) peptides. Positive responses were determined by Boolean gating with CD69+TFN-α+ and/or CD69+IFN-γ+.

To more closely investigate the long-term effects of Leronlimab, we collected blood samples at one timepoint per participant during study week 297–304. We detected integrated HIV-1 DNA in CD4+ T-cells from four of the five participants, with 01–038, the participant with the longest duration of HIV infection and most viral blips, harboring the greatest copy number of integrated HIV-1 DNA in CD4+ T-cells (Fig 2A). Thus, despite the presence of a latent HIV-1 reservoir in blood CD4+ T-cells, the absence of plasma viremia suggested that weekly Leronlimab was responsible for the maintenance of virologic suppression after ART interruption. Next, we performed mass cytometry (CyTOF) to profile 37 immune cell populations from participants’ PBMCs in comparison to healthy controls, which did not reveal any notable depletion of subsets of CD4+ T-cells, but did show decreases in T regulatory cells and increased monocytes compared to healthy controls (S2 and S3 Figs and S2 Table).

Because Leronlimab exerts its antiviral efficacy by outcompeting HIV for CCR5 binding, full CCR5 receptor occupancy by Leronlimab is required for a maximal antiviral response. To this end, we found that both cohorts had comparable mean (± SD) plasma concentrations of 48.6 (± 5.4) and 46.1 (± 27.0) μg/mL for cohort 1 and 2, respectively, likely due to the prolonged treatment period (Fig 2B). To quantify the saturation of cell surface CCR5 receptors by Leronlimab, we developed two complementary methods to measure free or Leronlimab-bound CCR5 receptors via flow cytometry (S4 Fig), which were used interchangeably to calculate for the percentage of CCR5 receptor occupancy (RO) by Leronlimab [17]. In all five participants, we observed near-complete CCR5 RO by Leronlimab on CD4+ T-cells, CD8+ T-cells, and CD14+ monocytes in the blood (Fig 2C). Together with the stringent viral control observed over seven years, these findings demonstrated that both doses of Leronlimab tested were sufficient to fully occupy CCR5 and protect CCR5+CD4+ T-cells from CCR5-tropic HIV-1 replication.

To evaluate if Leronlimab treatment would interfere with T-cell responses to HIV, we performed intracellular cytokine staining (ICS) on peripheral blood mononuclear cells (PBMC) using HIV and positive control human cytomegalovirus (HCMV) peptide antigens. Participants showed varying CD4+ memory T-cell responses to HIV despite being responsive to HCMV. In contrast, all participants showed distinct CD8+ memory T-cell responses (Fig 2D and 2E), confirming that long-term Leronlimab use did not abrogate cytotoxic T-cell responses to viral antigens.

Collectively, our findings demonstrated that Leronlimab treatment in humans was safe, well-tolerated, and maintained virologic suppression for over seven years. Weekly SC dose of 350 mg or 700 mg Leronlimab resulted in near-complete CCR5 RO on peripheral blood cells and suppression of CCR5-tropic HIV-1 replication, without depletion of immune system subsets or abolishment of antiviral cytotoxic T-cell immunity.

### Leronlimab treatment in SHIV-infected rhesus macaques for 12 weeks

**Fig 3:**
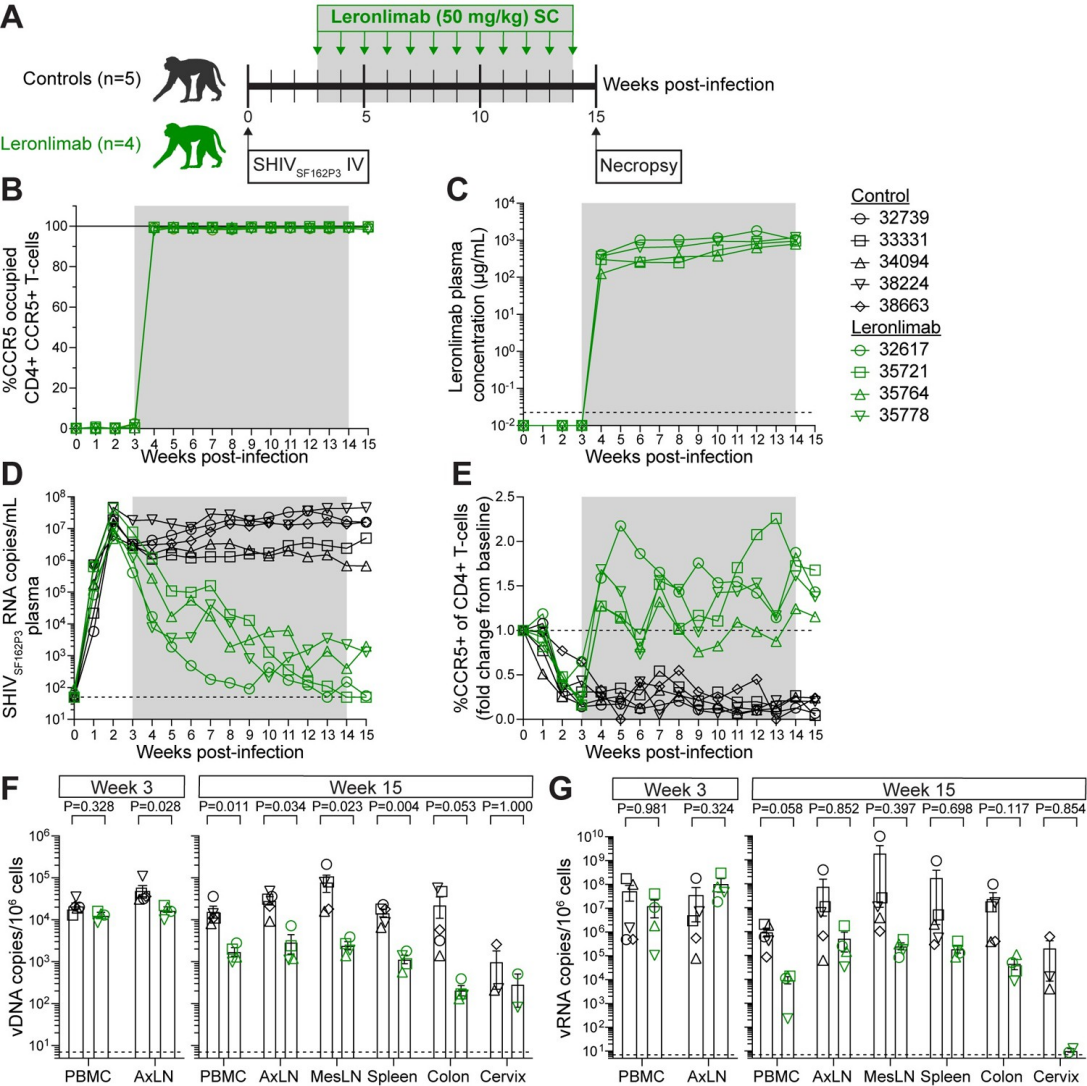
Leronlimab suppressed SHIV_SF162P3_ viremia in macaques. Macaques were infected intravenously with 1,000 TCID_50_ SHIV_SF162P3_. Leronlimab-treated group (n = 4, green) received weekly SC 50 mg/kg starting at week-3 post-infection and untreated controls (n = 5, black). **(A)** Study outline. **(B)** Longitudinal CCR5 RO levels by Leronlimab on blood CD4+CCR5+T-cell. **(C)** Longitudinal plasma concentration; horizontal dashed line denotes LOD (0.0226 μg/mL). **(D)** Longitudinal SHIV_SF162P3_ RNA copies/mL in plasma. Horizontal dashed line denotes LOD (50 copies/mL); undetectable viral loads graphed at LOD. **(E)** Longitudinal fold change from week-0 (baseline) for CD4+CCR5+ T-cell percentage in blood. (D-E) Weekly P-values can be found in S4 Table. **(F-G)** Mean (±SEM) cell-associated SHIV_SF162P3_ (F) vDNA and (G) vRNA at week-3 (pre-treatment) and week-15 (necropsy). Horizontal dashed lines denote LOD (7 copies/10^6^ cells). PBMC = peripheral blood mononuclear cell, AxLN = axillary lymph node, MesLN = mesenteric lymph node. (F-G) P-values calculated by two-way repeated-measures ANOVA with Tukey-Kramer adjustment. Gray box represents period of Leronlimab injections.

Experimental studies in non-human primates provide opportunities not possible in human clinical trials, including the ability to frequently and extensively sample anatomical sites of interest and synchronize infections [18]. We previously reported that Leronlimab, a humanized antibody, cross-reacted with macaque CCR5 [19]. To explore the impact of Leronlimab in acute infection, we intravenously infected nine macaques with 1,000 TCID_50_ of CCR5-tropic SHIV_SF162P3_. We selected SHIV_SF162P3_ for two reasons: 1) the parental Env, CCR5-tropic HIV-1_SF162_, is particularly resistant to CCR5-targeting agents [20] and 2) SHIV_SF162P3_ can switch tropism to use non-CCR5 coreceptors in macaques infection, which can help assess risk of viral escape [21]. SHIV_SF162P3_ infection proceeded without treatment for three weeks, after which four macaques received weekly SC injections of 50 mg/kg Leronlimab for 12 consecutive weeks. The remaining macaques (n = 5) served as untreated controls. At week 15, all animals were euthanized and anatomical tissues were collected for analysis (Fig 3A and S3 Table).

Consistent with Leronlimab treatment in humans, Leronlimab treatment in macaques was well-tolerated, with no major changes observed in longitudinal complete blood counts and serum chemistry parameters. However, absolute cell counts for lymphocytes, CD4+ T-cells, and CD8+ T-cells did trend higher in Leronlimab-treated macaques than in control macaques, as previously reported [13] (S5 and S6 Figs and S4 Table).

One week following the first Leronlimab dose, we observed near-complete CCR5 RO by Leronlimab on CD4+ T-cells and CD14+ monocytes from PBMC (Figs 3B and S7A) and axillary lymph node (S7B and S7C Fig), which was maintained throughout the study. The mean (± SD) Leronlimab plasma concentration was 702.3 (± 401.8) μg/mL (Fig 3C). Anti-drug antibody (ADA) responses against the humanized antibody, Leronlimab, may spontaneously develop in macaques, yet, we observed absent or minimal levels that did not impact plasma concentration or CCR5 RO (S7D Fig).

Prior to Leronlimab treatment, there was no statistically significant difference between control and Leronlimab-treated macaques for both peak and area-under-the-curve (AUC) plasma viral loads (S7E and S7F Fig). After receiving the second dose of Leronlimab, we observed a statistically significant reduction in weekly plasma viral loads in Leronlimab-treated macaques compared to controls, with viral loads that fell below the limit of detection in two of four macaques (Figs 3D and S7G and S4 Table). At the end of the study, the average plasma viral load for Leronlimab-treated macaques was 4.29 log_10_ lower than control macaques. Furthermore, we found no modifications within the Env V3 loop indicative of escape via CXCR4 usage, an alternative coreceptor for cell entry (S8 Fig).

Concomitant with near-complete CCR5 RO in the peripheral blood, Leronlimab-treated macaques exhibited a statistically significant resurgence in SHIV-susceptible CCR5+CD4+ T-cells, while control macaques experienced sharp and sustained decline in the same cell type (Fig 3E and S4 Table). Similarly, CD8+CCR5+ T-cells increased after Leronlimab treatment (S7H Fig), showing that Leronlimab did not deplete CCR5+ cells, likely due to the fact that IgG4 antibodies are essentially immunologically inert and exhibit low effector functions [22]. Recovery of CCR5+ T cells after Leronlimab treatment suggested that susceptible CCR5+ T cell populations were protected by Leronlimab during active viremia.

Next, we measured cell-associated SHIV DNA and RNA levels in multiple tissues. In line with plasma viral load results prior to Leronlimab treatment, viral DNA (vDNA) in PBMCs and viral RNA (vRNA) in PBMCs and axillary lymph nodes showed no significant difference between the two groups; however, vDNA in the axillary lymph nodes of control macaques were statistically higher than Leronlimab-treated macaques (Fig 3F and 3G). After 12 weekly Leronlimab treatments, we detected statistically lower vDNA in PBMCs, axillary and mesenteric lymph nodes, and spleen in the Leronlimab-treated group compared to the controls, with the colon approaching statistical significance (p = 0.053). While the vRNA levels in these tissues between the two groups did not reach statistical significance, levels were distinctly lower in the Leronlimab-treated animals than in control animals. Collectively, these results suggested that Leronlimab reduced infection of target CCR5+CD4+ T cells. This was in agreement with previous *in vitro* experiments where Leronlimab reduced viral replication in CD4+ T cells of rhesus macaque and human in a dose-dependent manner [10,19,20].

**Fig 4:**
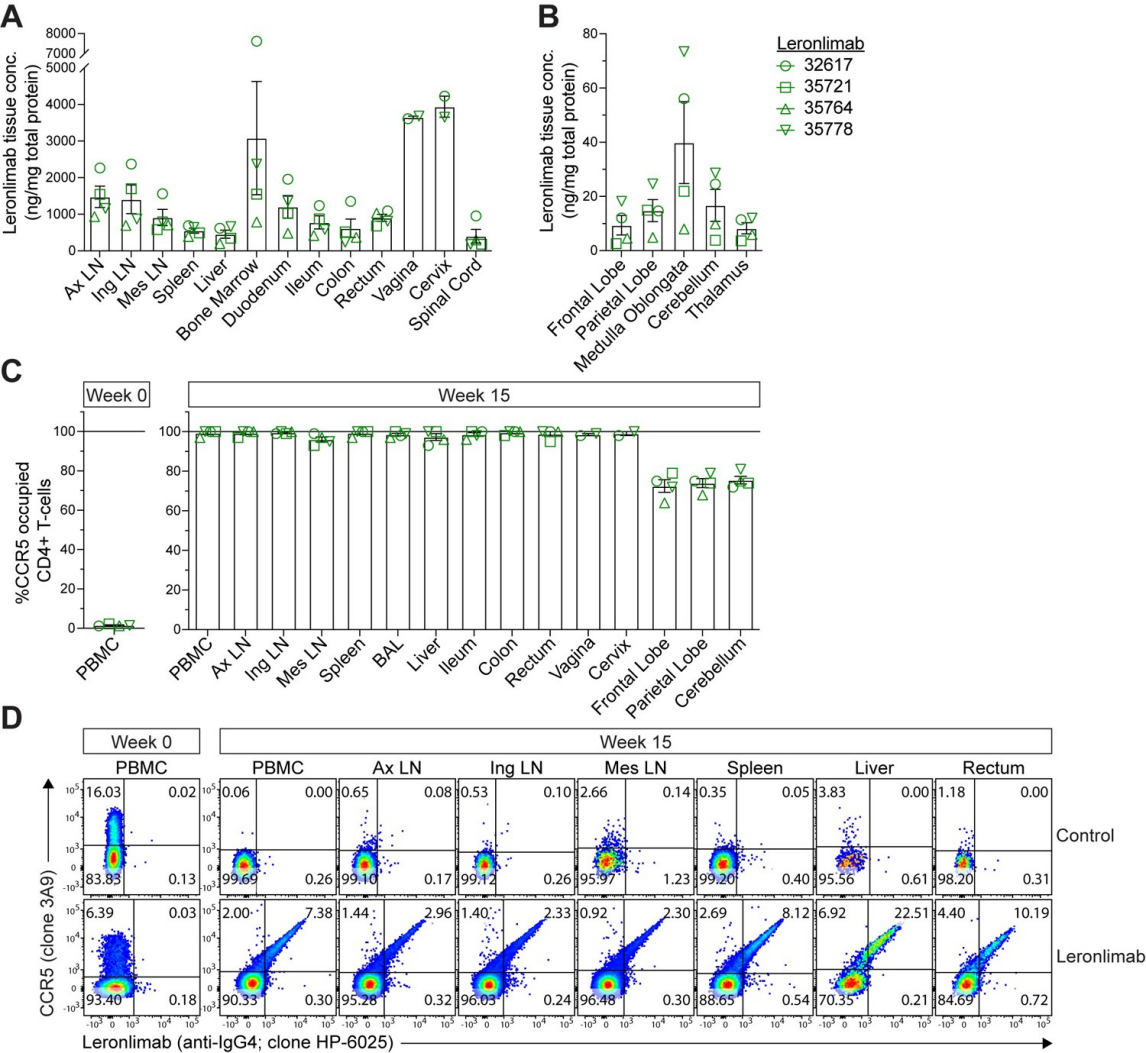
Tissue penetrance by Leronlimab. Tissues were collected at week-15 (necropsy). Tissue concentration in **A)** non-brain and **(B)** brain tissues. **(C)** CCR5 RO by Leronlimab on CD4+ T-cells. Panels A-C show mean (±SEM). **(D)** Representative flow cytometry plots showing the costaining of anti-CCR5 (clone 3A9) and Leronlimab (by anti-human IgG4, clone HP-6025) on CD4+ T-cells from control (38224) and Leronlimab-treated (35778) macaques. PBMC = peripheral blood mononuclear cell, Ax LN = axillary lymph node, Ing LN = inguinal lymph node, Mes LN = mesenteric lymph node, BAL = bronchoalveolar lavage.

Because ARVs tissue distribution is heterogenous [23,24], we investigated the degree of tissue penetrance by Leronlimab. During euthanasia at week 15, saline was perfused into Leronlimab-treated macaques to remove peripheral blood from tissues to allow for the assessment of tissue-resident Leronlimab. We detected a wide range of concentrations from all sampled tissues, and concomitantly measured near-complete CCR5 RO by Leronlimab in non-brain tissues. Leronlimab CCR5 RO in brain tissues was ~70%, with the lowest tissue concentration of all sampled tissues found in brain (Fig 4A, 4B, 4C and S5 Table). As expected, we found no Leronlimab bound to CCR5 receptors on tissue-resident CD4+ T-cells from control macaques (Figs 4D and S9). Noticeably, levels of CCR5+CD4+ T-cells were significantly lower in tissues from controls than Leronlimab-treated macaques due to SHIV-mediated depletion.

## Discussion

In summary, the studies described here of Leronlimab treatment in five chronically-infected humans and four acutely-infected macaques support the safety, efficacy, tolerability, and potency of Leronlimab as an anti-HIV therapeutic. Of note, the subgroup of five HIV+ participants presented here (of 41 subjects enrolled on Leronlimab monotherapy in the CD01 trial) have successfully suppressed HIV with Leronlimab for over seven years and these participants are currently continuing this treatment. Longitudinal plasma viremia in these participants was predominately below the limit of assay detection (40 copies/mL). However, it is important to note that participants on Leronlimab monotherapy exhibited a higher frequency of viral blips (7.1%) than those on combinational oral ART regimens (2.0%). Nevertheless, the ability of Leronlimab to sequentially control these viral blips demonstrated that Leronlimab-resistant variants did not arise during these seven years of Leronlimab monotherapy.

A limiting factor of the clinical study presented here is the small sample size. However, the significance of this study comes from the greater than seven-year duration of Leronlimab monotherapy treatment, which, to our knowledge, is the longest antibody treatment for HIV reported to date. These successful proof-of-concept results for multiyear antibody monotherapy treatment for HIV aligns with the idea that small sample sizes are appropriate for innovative and translational research of new approaches [25]. Nevertheless, beyond these five participants, larger studies of Leronlimab monotherapy are required to determine if this therapeutic modality is sufficiently efficacious in the highly heterogenous population of HIV+ individuals worldwide. Indeed, larger clinical studies on this topic are currently underway, although these cohorts are treated for much shorter durations than seven years.

In the macaque study, 12 weekly 50 mg/kg Leronlimab doses effectively reduced plasma viremia by 10,000-fold compared to control macaques. Interestingly, the viral load set points for untreated macaques in our study were significantly higher than previously reported for SHIV_SF162P3_ infection in rhesus macaques [26], likely due to the high IV dose utilized (1,000 TCID_50_). These higher levels of set point viremia and reservoir seeding provided stricter conditions to study the potency and efficacy of Leronlimab, and suggest that this dose may be ideal for future macaque studies utilizing SHIV_SF162P3_ to assess cure-based regimens. In agreement with Leronlimab use in humans, we found that short-term use of Leronlimab in macaques did not cause emergence of CXCR4-tropic viral variants. More importantly, the use of macaques provided critical insights on tissue distribution that would not be possible to measure in human participants. Using macaques, we detected Leronlimab at varying concentrations in all examined tissues, which resulted in saturated binding of CCR5 on CD4+ T cells in non-CNS tissues. Because mucosal and lymphatic tissues are anatomical sites for early viral replication after transmission and major reservoir depots during suppressive ART [23,27,28], the high penetrance and binding of Leronlimab to CCR5 on CD4+ T cells in these tissues suggests Leronlimab could have applications for HIV PrEP [19] and cure.

In both the human and macaque studies, Leronlimab monotherapy did not drive viral escape via conversion of CCR5-tropic to CXCR4-tropic viruses. Moreover, in the 44% of participants who were not able to maintain virologic suppression in the original CD01 study, the majority showed a lack of coreceptor switching by standard viral tropism assays (Trofile DNA Assay and PhenoSense Entry), even though two participants were believed to have preexisting CXCR4-tropic latent virus. While the presence of dual-tropic viruses is a risk for anti-CCR5 therapeutics, there may be a high genetic cost to using CXCR4. This was observed in the Berlin Patient, the first functionally cured HIV individual, who was cured after undergoing hematopoietic stem cell transplantation (HSCT) using donor cells homozygous for a 32-base pair deletion in *ccr5* [29]. Prior to HSCT, the latent viral pool was sequenced and found to contain a small percentage (2.9%) of CXCR4-tropic viruses. However, no outgrowth of CXCR4-tropic viruses was observed despite showing normal levels of CXCR4+ T cells after HSCT [29]. Various functional *in vitro* tropism assays demonstrated that only the patient-derived CCR5-tropic strains, and not the CXCR4-tropic strains, were infectious [30]. Moreover, upon discontinuation of ART treatment in long-term suppressed individuals, all rebounding viruses assayed still utilized CCR5 as the coreceptor for infection [31], demonstrating a dependence on CCR5 despite the presence of dual-tropic variants and high *env* diversity in the latent viral population.

There are many potential benefits for the use of antibody-based drugs, including long-acting formulations to allow for infrequent dosing, higher target specificity to decrease side effects, and enhanced tissue penetrance in gastrointestinal tissues not seen with current ARVs [24,32]. A switch from daily oral pills to weekly or monthly antibody injections would substantially improve user experience and lower adherence-associated risks of drug-resistance. Currently, maraviroc is the only FDA-approved CCR5 inhibitor for HIV. In a 10-day monotherapy study, twice-daily 300 mg maraviroc reduced viral load by 1.84 log_10_ [33]. A similar reduction of 1.83 log_10_ was observed from a single Leronlimab injection of 5 or 10 mg/kg [13,15], which is the approximate dosage range used to treat participants in our study based on their individual average weights (4.34–11.76 mg/kg). While both drugs are CCR5-targeting and show similar efficacy in short-term use, the difference in their mechanism of action allows maraviroc-resistant HIV to continue infecting maraviroc-occupied CD4+CCR5+ T-cells [12], perpetuating the development of ARV resistance. In contrast, previous studies demonstrate the ability for Leronlimab to prevent viral replication *in vitro* [10,19,20] while the *in vivo* human and macaque studies described here support the high genetic barrier to resistance and potency of Leronlimab. Collectively, these findings suggest that Leronlimab is a promising entry inhibitor candidate. Future studies are warranted to expand and characterize the use of Leronlimab as an HIV therapeutic.

## Materials and methods

### Ethics statement

#### Human subjects research

Studies were approved by Western Institutional Review Board (WIRB) (WIRB Protocol Numbers: 20140327 and 20142157). Declaration of Helsinki and the International Conference on Harmonization Good Clinical Practice Guideline were used to conduct studies. Trials took placed at the Quest Clinical Research in San Francisco, California (ClinicalTrials.gov Identifiers: NCT02175680 and NCT02355184). All participants provided written informed consent prior to inclusion in studies.

Human studies were an open-label, single arm, phase 2b clinical trials to evaluate the efficacy, safety, and tolerability of Leronlimab (Vyrologix) monotherapy for maintenance of viral suppression in subjects who were stable on combination antiretroviral therapy (Table 1). HIV-infected participants of all sexes were eligible if they were older than 18 years of age, were stable on antiretroviral therapy for the last 12 months, had two or more potential alternative ART regiment options, and had nadir CD4 T-cell counts of > 200 cells/mm^3^. At the Screening Visit, eligible participants had exclusive CCR5-tropic virus as determined by Trofile DNA assay and no documented detectable viral loads (<50 HIV-1 RNA copies/mL) within the last 12 months. Participants with CXCR4-tropic or dual-mixed tropic viruses, hepatitis B infection, AIDS-defining illness, and history of using entry, attachment, CCR5 co-receptor, or fusion inhibitors were excluded from the study.

Leronlimab was supplied by CytoDyn, Inc. Eligible participants received weekly SC injections of 350 mg Leronlimab (NCT02175680) for 12 weeks. Total treatment duration was 14 weeks, where participants had a one week overlap with existing ART regiment at the start and conclusion of study. After which, consenting participants continued weekly SC injections for at least 160 weeks (NCT02355184) (Fig 1A). Plasma HIV-1 RNA levels were measured using a quantitative assay (*Abbott Real time*), where the lower limit of detection was 40 copies/mL. Participants were monitored weekly during the first 12 weeks, once-every-two weeks from study weeks 12–52, and monthly thereafter. CD4+ T-cells counts were monitored using the TruCount Assay (LabCorp). Participants re-initiated their previous ART regiment if plasma HIV-1 RNA level was >400 copies/mL on two consecutive blood draws at least three days apart.

#### Animal research

Rhesus macaque care plan and all experimental protocols, procedures, and administered reagents were approved by the Oregon National Primate Research Center (ONPRC) Institutional Animal Care and Use Committee (IACUC). ONPRC is a Category I facility. The Laboratory Animal Care and Use Program are fully accredited by the American Association for Accreditation of Laboratory Animal Care (AAALAC), with an approved Assurance (#A3304-01) for the use and care of animals on file with the NIH Office of Laboratory Animal Welfare. The ONPRC IACUC adheres to national guidelines established in the Animal Welfare Act (7 U.S.C. Sections 2131–2159) and the Guide for the Care and Use of Laboratory Animals (8th Edition) as mandated by the U.S. Public Health Service Policy. As well as using the standards of the US NIH *Guide for the Care and Use of Laboratory Animals* (National Academies Press, 2011).

Macaques (*Macaca mulatta)* used in this study were housed at the ONPRC in Animal Biosafety level (ABSL)-2+ rooms with autonomously controlled temperature, humidity, and lighting. At assignment, macaques were free of *Cercopithecine* herpesvirus 1, D-type simian retrovirus, simian T-lymphotrophic virus type 1, and *Mycobacterium tuberculosis*. Macaques were typed for the MHC alleles Mamu-B*17, and Mamu-B*08. Macaques positive for Mamu-B*17 and/or B*08 were excluded when possible or placed into the control group when it was not possible to exclude biased results. All attempts were made to pair house macaques during the study period by gender and treatment group. When compatible partners were not available, macaques had auditory, visual, and olfactory contact with neighboring macaques and were provided with an enhanced enrichment plan designed and overseen by a macaque behavioral specialist. Macaques were fed Purina LabDiet 5000 (Purina Mills International, St. Louis, MO) twice daily and received daily food enrichment (e.g., fresh fruit, vegetables). Automatic water systems provided fresh and potable water. Ketamine HCl (Ketathesia, Henry Schein Animal Health) with or without Dexmedetomidine (Dexmedesed, Dechra, Overland Park, KS) was used to sedate macaques for procedures, including subcutaneous injections of Leronlimab, venipuncture, tissue biopsy, and SHIV challenge. At the study endpoint, macaques were euthanized with sodium pentobarbital overdose (>50 mg/kg) and exsanguinated via the distal aorta. Aboard certified veterinary pathologist performed and collected tissues at necropsy.

Nine adult macaques were used in the study, with five macaques in the control group and four macaques in the Leronlimab-treatment group. See S3 Table for the ID number, gender, age, weight, and MHC alleles of these animals. Animals were balanced between the control and Leronlimab-treatment groups by gender. At the beginning of the study, all macaques were intravenously infected with 1,000 TCID_50_ of the SHIV_SF162P3_ stock provided by Nancy Haigwood. [34]. Macaques received no treatments until study week-3, after which, Leronlimab-treated macaques (n = 4) initiated weekly SC 50 mg/kg Leronlimab for 12 consecutive weeks, while untreated control macaques (n = 5) received no treatments. Sites for the Leronlimab injection alternated every three weeks between the dorsal scapular and dorsal thoracic areas. At week-15, animals were euthanized and tissues were collected and processed, as described below.

#### Mass cytometry (CyTOF)

Mass cytometry was performed as previously described [35]. Briefly, Maxpar Direct Immune Profiling Assay reagents were purchased from Fluidigm Inc. Cryopreserved peripheral blood mononuclear cells from each of the human participants receiving Leronlimab during study week 297–304 were analyzed. Live cells were blocked with Human TruStain FcX for 10 minutes, then stained with a panel of metal-labeled antibodies against 30 cell surface markers (S6 Table) for 30 minutes on ice and then washed twice. Stained cells were then fixed with fresh 1.6% formaldehyde for 10 minutes, aspirated, and stained with intercalator-IR. Samples were acquired on a Helios Mass Cytometer and resulting FCS files normalized using a bead-based normalization algorithm and analyzed with Maxpar Pathsetter software.

#### T-cell assays

T-cell responses in PBMC were measured by flow cytometric intracellular cytokine staining (ICS) as previously described [36]. One million PBMC were incubated with overlapping 15-mer peptide pools spanning HIV-M (subtype B) Gag or Nef, and co-stimulated with CD28 and CD49d antibodies (BD Biosciences) for 1 hour, followed by incubation with Brefeldin A (Sigma-Aldrich) for 8 hours. Similarly, stimulation using human cytomegalovirus (HCMV) 15-mer peptide pools spanning IE1 and pp65 served as positive controls, while no antigen incubation served as background controls. Cells were surface stained with antibodies against CD3, CD4, CD8, CD95, CD28, and amine-reactive dye, and then fixed with 2% paraformaldehyde (PFA). BD FACS Permeabilizing Solution (BD Biosciences) was used to permeabilize the membranes before staining intracellularly for IFN-γ, TNF-α, and CD69. Samples were collected on a BDLSR-II instrument with FACsDIVA version 6.1, and analyzed with FlowJo v10 (Tree Star) by gating on singlet, live, CD3+, CD4+ or CD8+ cells. Positive responding CD4+ and CD8+ memory (CD95+) T-cells were measured by Boolean gating on CD69+/TFN-α+ and/or CD69+/IFN-γ+ cells. Presented as HIV positive responses were sums of individual HIV Gag and Nef responses, while positive HCMV responses were sums of individual HCMV IE1 and pp65 responses.

#### SHIV stock

SHIV_SF162P3_ stock (day 7 post-infection harvest, dated 03-17-17, 170 ng/mL p27 by ELISA, and 6,68x10^5^ TCID_50_/mL in TZM-bl cells) was generated, characterized, and kindly provided by Nancy Haigwood [34].

#### Processing blood and tissue

Whole blood for complete blood count (CBC), plasma, and peripheral blood mononuclear cell (PBMC) processing was collected with EDTA-treated tubes (BD Biosciences). CBC was measured using an ABX Pentra 60 C+ Hematology Analyzer. PBMC and plasma were isolated from whole blood by density gradient centrifugation using Ficoll-Hypaque (Sigma Aldrich) by spinning at 1,860 x g for 30 minutes. Plasma was isolated from the top layer while PBMC was isolated from the buffy coat and washed once with R10 (RPMI 1640 containing 10% fetal bovine serum (FBS)). Whole blood for serum chemistry analysis was collected in non-anticoagulant tubes and spun down at 1,860 x g for 10 minutes to separate serum from clotted blood. Serum chemistry levels were assessed using an ABX Pentra 400 Chemistry Analyzer.

Lymph nodes and spleen were collected in R10, diced into fine pieces with a scalpel, and forced through a 70-μm cell strainer into R10 to collect single cell suspensions. Red blood cells from spleen-derived single cell suspensions were lysed using the ACK lysis buffer (ThermoFisher) following the manufacturer’s protocols. Bronchoalveolar lavage fluid was collected in phosphate-buffered saline (PBS; Fisher Scientific) and filtered through a 70-μm cell strainer.

Gastrointestinal tissues were collected in R10, diced into fine pieces by a scalpel, placed into a 50 mL conical tube containing R3 (RPMI 1640 containing 3% FBS) with 0.5 M EDTA, and incubated in 37°C with 250 rpm shaking for 30 minutes to remove mucus coating. Afterward, tissues were poured over a tea strainer to remove EDTA and mucus coating, and then sequentially washed three times with Hank’s Buffered Salt Solution (HBSS; Fisher Scientific) by pouring over a tea strainer between each wash. EDTA-free tissues were placed into a new 50 mL conical tube containing Digestion Media (R3, 0.2 mg/mL collagenase (Sigma-Aldrich, St. Louis, MO) and 0.2 mg/mL DNase I (Roche, Indianapolis, IN)), and then incubated in 37°C with 250 rpm shaking for 1 hour. Digested tissues were filtered through a 70-μm cell strainer and spun down at 830 x g for 4 minutes to pellet the cells. The pellet was resuspended in 70% Percoll (Cytiva) and underlaid in 37% Percoll before spinning at 500 x g for 20 minutes. Single cell lymphocytes were collected from the center interface into R10 and stained.

Rectum, cervix, and vagina were processed with the gastrointestinal tissue protocol described above, with the following change: HBSS was used in replacement to R3 with 0.5 M EDTA during the first 30 minutes incubation step. As well, the liver was processed with the gastrointestinal tissue protocol, with the following change: exclusion of the first 30 minutes incubation step. Finally, brain regions were processed with the gastrointestinal tissue protocol described above, with the following changes: 1) exclusion of the first 30 minutes incubation step and 2) incubation of tissues in Digestion Media at 37°C without shaking.

#### Leronlimab CCR5 receptor occupancy (RO)

Two methods were used and validated to measure the percentage of CCR5 RO by Leronlimab on the cell surface (S4 Fig). Eq 1 measured bound CCR5 receptors by using anti-human IgG4 to directly measure Leronlimab-occupied CCR5 receptors, while Eq 2 measured unoccupied CCR5 receptors by using Pacific Blue-conjugated Leronlimab (termed Leronlimab-PB). These two complementary methods were based on RO assays for anti-PD-1 antibodies in clinical trials [37].


$$\%\;RO=\frac{\%\;IgG4}{\%\;Saturated\;IgG4}x\;100\%$$ 1

From CCR5+ (measured by clone 3A9) cells, RO for Eq 1 was defined by the percentage of Leronlimab+ (measured by anti-human IgG4) divided by the percentage of Leronlimab+ following incubation with a saturating concentration of unlabeled Leronlimab. To perform the staining, 0.3–1 x 10^6^ PBMCs or single cells from tissue homogenates were incubated with or without 5 μg/mL of unlabeled Leronlimab to achieve Leronlimab saturation of cell surface CCR5 receptors for 30 minutes at room temperature (RT) in the dark. Cells were washed once with PBS, spun down at 830 x g for 4 minutes, aspirated, and stained with anti-human IgG4 for 30 minutes at RT in the dark. After washing once with FACS buffer (PBS with 10% FBS) and once with PBS, cells were stained with CD45, CD3, CD4, CD8, amine-reactive dye, and CCR5 (via antibody clone 3A9) for 30 minutes at RT in the dark. Finally, cells were washed twice with PBS and fixed with 2% PFA for more than 30 minutes at 4°C before collecting on LSR-II instrument and FACsDIVA version 6.1. Using FlowJo v10 (Tree Star), cells were gated on CD45, singlet, live, CD3+, CD4+, CCR5+, and anti-human IgG4+.


$$\%\;RO=\frac{\%\;IgG4}{\%\;IgG4+\%\;Leronlimab-PB}x\;100\%$$ 2

From CCR5+ (measured by clone 3A9) cells, RO for Eq 2 was defined as the percentages of cells positive for Leronlimab (measured by anti-human IgG4) divided by the sum of anti-human IgG4 and saturating concentration of Leronlimab conjugated to Pacific Blue (termed Leronlimab-PB), as previously described [19]. Briefly, 0.3–1 x 10^6^ PBMC or single cells from tissue homogenates were washed with 10% mouse serum (Fisher Scientific) in PBS and then incubated with 100 μL of 100% mouse serum for 1 hour at RT in the dark to block nonspecific Fc receptors binding to anti-human IgG4 antibodies by monocytes. Cells were washed with 10% mouse serum, replenished with new 100 μL of 100% mouse serum, and incubated with anti-human IgG4 for 30 minutes at RT in the dark. Next, cells were washed once with FACS buffer and four times with PBS. Cells were stained with CD45, CD3, CD4, CD8, CD16, CD14, amine-reactive dye, CCR5 (via antibody clone 3A9), and Leronlimab-PB for 30 minutes at RT in the dark. Lastly, cells were washed twice with PBS and fixed with 2% PFA for more than 30 minutes before collecting on LSR-II instrument and FACsDIVA version 6.1. Using FlowJo v10 (Tree Star), cells were gated on CD45+, singlet, live, CD3+, CD4+, and CCR5+ events. The CD4+ CCR5+ population was further gated on anti-human IgG4+ or Leronlimab-PB+ events.

### Measurement of Leronlimab concentration

Enzyme-linked immunosorbent assay (ELISA) was used to detect Leronlimab in plasma and tissues as previously described [19]. Briefly, anti-idiotype antibody PA22 (CytoDyn, Vancouver, WA) was used to coat half-area 96-week Costart Assay Plates (Corning) at 1.5 μg/mL in carbonate-bicarbonate buffer (ThermoFisher) and incubated overnight at 4°C. The next day, plates were washed three times with PBS-T (PBS with 0.1% Tween-20) and blocked with Blocking Buffer (PBS with 0.4% Tween-20 and 10% bovine serum albumin (Sigma)) for at least two hours in RT. A standard curve was generated with serial Leronlimab dilutions that ranged from 4.7–300 ng/mL. Plasma samples were first heat-inactivated for 30 minutes at 56°C and spun for 20 minutes at 12,000 x g to pellet residual debris. Supernatants were used for ELISA by diluting in Blocking Buffer with at most four appropriate dilutions to assay. Blocked plates were washed three times with PBS-T. Diluted plasma and standard samples were added into the plates in duplicates and incubated for 30 minutes in RT. Afterward, plates were washed three times with 0.5 M NaCl in PBS. Mouse anti-human IgG4 pFc’-horseradish peroxidase (Southern Biotech) was diluted 20,000-fold with Blocking Buffer, added to the plates, and incubated for 30 minutes in RT. Plates were finally washed three times with PBS-T and developed for two minutes with 3,3’,5,5’-Tetramethylbenzidine (TMB) substrate (Southern Biotech). Reactions were stopped with 1 N H_2_SO_4_. Plates were read on the Synergy HTX Milti-Mode Microplate Reader (BioTek) and Gen5 v3.10 at two absorbance wavelengths: 650 nm for the developing reaction and 450 nm for the developed reaction. The final OD used for calculation was obtained by subtracting OD_450 nm_ with OD_650 nm_. Plasma concentration (μg/mL) was determined using the generated standard curve, with an assay limit of detection of 0.0226 μg/mL.

To quantify Leronlimab concentration in tissues, 2–5 mm^2^ pieces were placed into Lysing Matrix tubes (MP Biomedicals), with 200–500 μL (adjust volume based on tissue size) of complete EDTA-free Protease Inhibitor Cocktail (Sigma Aldrich) in PBS. Tissue was disrupted by beating in a Beadbeater (Biospec) device by three cycles of 1 minute beating and 1 minute on ice. Supernatant from the tissue homogenate was transferred into a Sarstedt tube and spun for 20 minutes at 12,000 x g to pellet residual debris. Protein-containing supernatant was transferred into a new Sarstedt tube and stored at -80°C until assayed. Leronlimab concentration in the tissue, presented as ng of Leronlimab to mg of total protein, required two ELISAs to determine 1) Leronlimab concentration as described above and 2) total protein concentration measured with Pierce Coomassie Plus Broadford Assay (ThermoFisher) following manufacturer’s instructions.

### Measurement of anti-drug antibody (ADA) against Leronlimab

ELISA was used to detect ADA against Leronlimab in plasma as previously described [19]. Briefly, 2 μg/mL Leronlimab (Cytodyn, Vancouver, WA) was diluted in carbonate-bicarbonate buffer (ThermoFisher) and coated into half-area 96-well Costar Assay Plates (Corning) overnight at 4°C. The next day, plates were washed three times with PBS-T and blocked with Blocking Buffer for at least two hours in RT. Afterward, blocked plates were washed three times with PBS-T. Heat-inactivated plasma samples were serially diluted in Blocking Buffer with six serial dilutions and plated onto blocked plates in duplicates for 30 minutes in RT. After this incubation step, plates were washed three times with 0.5 M NaCl in PBS and incubated with 5,000-fold diluted secondary antibody that recognized rhesus macaque IgG and conjugated to HRP (anti-rhesus IgG1/3[1B3]-HRP; NHP Reagent Resource; 0.5 mg/mL) in Blocking Buffer. Plates were incubated at RT for 30 minutes, with the remaining steps following the quantification ELISA described above. ADA titers were defined as the reciprocal of the highest dilution of the sample that yielded a positive result (*e*.*g*., dilution of 1/2460 = titer of 2460). A positive result was defined as twice that of background value for each individual macaque.

### CCR5 genotyping

For CCR5 sequencing, genomic DNA was extracted from subject PBMC using DNeasy blood and tissue kit (Qiagen). The CCR5 region from 5’ UTR through coding sequence stop codon was amplified with 2X Phusion mastermix (New England Biolabs) using forward primer (5’-TCATGTGGAAAATTTCTCATAGCTTCAGA-3’) and reverse primer (5’-CGAGTAGCAGATGACCATGACAA-3’) with the following PCR conditions: 98C for 30 seconds, [98C for 15 seconds, 56C for 20 seconds, 72C for 4 minutes] x 40 cycles, 72C for 3 minutes, 4C. The resulting ~3.5kb PCR amplicons were gel-purified using a 1% agarose gel and NucleoSpin Gel and PCR Clean-up Kit (Macherey-Nagel). Gel-purified amplicons were prepared for sequencing using Nextera XT DNA Library Prepartion kit (Illumina) according to manufacturer’s instructions. Libraries were sequenced in parallel on the Illumina MiSeq according to manufacturer’s instructions. Sequences (>80,000 reads per subject) were aligned to CCR5 (NC_000003.12:46370142–46376206) and analyzed for SNPs and delta32 genotype described by Mummidi *et al* [38]. Haplotypes defined by Mummidi *et al*. were called for each subject based on sequence data.

### Viral nucleic acid detection

Detection of integrated HIV-1 DNA was performed as previously described [35,39]. Briefly, HIV-1 viral DNA was isolated from PBMCs and enriched for CD4+ T-cells to purities of more than 95% with an EasyStep Human CD4+ T-cell enrichment kit (Stemcell Technologies). DNA was extracted from enriched CD4+ T-cells using AllPreP DNA/RNA/miRNA Universal Kit (Qiagen) and measured by a ND-2000 spectrophotometer (NanoDrop Technologies) for purity and concentration. To determine integrated HIV-1 DNA copies in CD4+ T-cells, DNA samples were first pre-amplified using LTR-Alu specific primers ULF1, Alu1 (5’-TCCCAGCTACTGGGGAGGCTGAGG-3’) and Alu2 (5’-GCCTCCCAAAGTGCTGGGATTACAG-3’). Pre-amplification was performed in 50 μL reaction volumes using 5 μL Taq Buffer, 3 μL of MgCl_2_, 0.6 μL of each dNTP, 1.5 μL of each primer, 0.5 μL of Taq polymerase, and 5 μL of DNA template. Reactions were performed on a GeneAmp PCR System 9700 (Applied Biosystems Inc.) with the following thermal conditions: 95°C for 8 minutes; [95°C for 1 minute, 55°C for 1 minute, 72°C for 15 minutes] x 12 cycles. Integrated HIV-1 DNA samples were quantified with a qPCR TaqMan assay using Lambda T (5’-ATGCCACGTAAGCGAAACT-3’) and UR2 (5’-CTGAGGGATCTCTAGTTACC-3’; HXB2 583–602), and coupled with a FAM-BHQ probe (5’-CACTCAAGGCAAGCTTTATTGAGG-3’). The reaction volume was 20 μL, containing 10 μL of 2x TaqMan Universal Master Mix II including UNG (Life technologies), 0.25 μL of 100 μM per primer, 0.4 μL of 10 μM probe, and 5 μL pre-amplified PCR product (1:10 dilution). Reactions were performed on a StepOne Plus Real-time PCR System (Applied Biosystems Inc) with the following thermal conditions: 50°C for 2 minutes; 95°C for 10 minutes; [95°C for 15 seconds and 60°C for 1 minute] x 60 cycles. A standard curve was created with the CD3 gene, which allowed for the calculation of cell-equivalent DNA copy numbers.

Detection of SHIV nucleic acid was conducted previously described [19,40]. Briefly, all SHIV nucleic acid detection assays were performed by members of the ONPRC Molecular Virology Core, who were blinded in the treatment conditions of each animal for all time points. Maxwell 16 instrument (Promega, Madison, WI) was used to extract nucleic acid from plasma and PBMC cells using LEV Viral Total Nucleic Acid Kit and LEV Whole Blood Nucleic Acid kit, respectively, following manufacturer’s protocols. Tissues were disrupted in Tri-reagent, and extraction of DNA and RNA was performed as previously described [19,36]. Viral copies were measured by RT-qPCR or qPCR that targeted a highly conserved sequence of Gag by using the SGAG21 forward primer (5’-GTCTGCGTCATPTGGTGCATTC-3’), SGAG22 reverse primer (5’-CACTAGKTGTCTCTGCACTATPTGTTTTG-3’), and pSGAG23 probe (5′-6-carboxyfluorescein [FAM]-CTTCPTCAGTKTGTTTCACTTTCTCTTCTGCG-black hole quencher [BHQ1]-3′).

RT-qPCR reactions were performed to quantitate SHIV viral RNA copies in plasma. The reactions used the TaqMan Fast Virus 1-Step Master Mix (Applied Biosystems), all RNA extracted from 300 μL of plasma, 900 nM SGAG21, 900 nM of SGAG22, and 250 nM pSGAG23. A standard curve was created by using in vitro transcribed SIVgag RNA serially diluted in 5 ng/μL yeast tRNA (Sigma R5636), with known SIV-positive plasma RNA and nuclease-free water serving as the positive and negative controls, respectively. Applied Biosystems QuantStudio 6 Flex instrument (Life Technologies) was used to run the reactions, with the following thermal conditions: 50°C for 5 minutes; 95°C for 20 seconds; [95°C for 3 seconds, 60°C for 30 seconds] x 45 cycles. The limit of detection for this assay is 50 copies/mL.

To detect SHIV viral RNA copies in cell pellet and tissue samples, 2.5 μg of extracted RNA samples were synthesized to complementary DNA (cDNA) using 20 U Superscript II RT (ThermoFisher/Invitrogen), 5 mM MgCl_2_, 0.5 mM dNTPs, 1 mM dithithreitol (DTT), 150 ng random hexamers, 1x TaqMan PCR buffer (with 0.05% gelatin and 0.02% Tween-20), and 20 U RNAaseOut for a final volume of 30 μL. Reactions were ran using the following thermal conditions: 25°C for 15 minutes, 42°C for 40 minutes, 90°C for 15 minutes, 25°C for 30 minutes, and 4°C hold. Using the entire RT-PCR reactions, qPCR reactions were performed by adding 1.25 U Platinum Taq (Applied Biosystems), 600 nM of SGAG21, 600 nM of SGAG22, 100 nM pSGAG23, 1x TaqMan PCR II buffer, 4.5 mM MgCl_2_, and 50 nM ROX passive reference dye for a final volume of 50 μL. A standard curve was created by using in vitro transcribed SIVgag RNA serially diluted in 100 ng/μL yeast tRNA (Sigma R5636). qPCR reactions were performed in an Applied Biosystems ABI 7500 instrument using the following thermal conditions: 95°C for 2 minutes; [95°C for 15 seconds, 60°C for 1 minute] x 45 cycles.

To detect SHIV viral DNA copies in cell pellet and tissue samples, 2.5 μg of extracted DNA samples were first heated at 95°C for 5 minutes, placed on ice to chill, and then added to Taqman Fast Advanced Master Mix (Life Technologies), 600 nM of SGAG21, 600 nM of SGAG22, and 100 nM pSGAG23. A standard curve was created by serial dilutions of a linearized plasmid containing the SIV gag sequencing using TE buffer that contained 2.5 ng/mL SIV-negative rhesus macaque genomic DNA. Applied Biosystems QuantStudio 6 Flex instrument (Life Technologies) was used to run qPCR reactions with the following thermal conditions: 50°C for 2 minutes; 95°C for 20 seconds; [95°C for 1 second, 60°C for 20 seconds] x 45 cycles. The limit of detections for this assay are 10 copies/million cells for cell pellets and 7 copies/million cells for tissue biopsies.

### Env sequencing

SHIV_SF162P3_ Env sequencing and analysis were performed as previously described [41–45]. Briefly, QIAamp MinElute Virus Spin Kit (Qiagen) was used to extract viral RNA from viral stock and plasma samples following manufacturer’s instructions. Complementary DNA was generated with the SuperScript III One-Step RT-PCR with Platinum Taq (ThermoFisher) with the following primers: SHIV *env* Forward primer (GGCATAGCCTCATAAAATATCTG) and SHIV *env* Reverse primer (ACAGAGCGAAATGCAGTGATATT), giving a ~4.5kb amplicon of *env*. Eppendorf Mastercycler Pro S Thermal Cyclers was used to run the RT-PCR reaction, using the following thermal conditions: 50°C for 30 min; 94°C for 2 min; [94°C for 15 sec, 60°C for 1 min, 68°C for 4 min] x 2 cycles; [94°C for 15 sec, 58°C for 1 min, 68°C for 4 min] x 2 cycles; [94°C for 15 sec, 60°C for 1 min, 68°C for 4 min] x 45 cycles; 68°C for 10 min; and held at 4°C. PCR amplicons were purified using 1% agarose gels and NucleoSpin Gel and PCR Clean-up Kit (Macherey-Nagel). Following manufacturer’s instructions, Nextera XT DNA Sample Prep Kit (Illumina) was used to generate dual-indexed Illumina MiSeq-compatible libraries and then purified with AMPure XP magnetic beads (Beckman Coulter). Dual-indexed libraries were analyzed on an Agilent 2100 Bioanalyzer using the HS DNA kit (Agilent) before normalizing to 2 nM and pooling to an equimolar ratio for Illumina MiSeq run. Sequences were processed as previously described [41,42], where Trimmomatic version 0.39 and BWA-MEM version 0.7.17-r1188 [43,44] were used to trim the raw data and align to the consensus SHIV_SF162_ sequence (GenBank Accession No. KF042063.1). Deletion and insertion polymorphisms and single nucleotide polymorphisms (SNPs) were called for bases with a quality score above 17. Identity of the associated read was retained for each SNP, allowing the phase of SNPs to be considered. This allows for amino acid translations to be calculated based on the sequence per individual read rather than on the consensus SHIV_SF162_ sequence. SequenceAnalysis module, written by LabKey Server 21.3 [45] was used to analyze SNPs and visualize mutations.

### Antibodies

The following conjugated antibodies were used in these studies: a) from BD Biosciences, D058-1283 (CD45; PE Cy7; cat# 561294), SP34-2 (CD3; Alexa 700; cat# 557917), SP34-2 (CD3; PE; cat# 552127), LP200 (CD4; PerCP-Cy5.5; cat# 552838), RPA-T8 (CD8; PacBlu; cat# 558207), SK1 (CD8; TruRed; cat# 341051) 3GB (CD16; Alexa 700; cat# 560713), 25723.11 (IFN- γ; APC; cat# 502512), 6.7 (TNF-α; PE; cat# 554513), 3A9 (CCR5; APC; cat# 560748), SK1 (CD8; BUV737; cat# 612754), L200 (CD4; BUV395; cat# 564107), FN50 (CD69; PE-Texas Red; cat# 562617), SP34-2 (CD3; Pacific Blue; cat# 558124); b) from BioLegend, OKT4 (CD4; APC-Cy7; cat# 305612), RPA-T4 (CD4; APC; cat# 300537); c) from Beckman Coulter, RMO52 (CD14; PE-Texas Red; cat# IM2707U); d) from Sigma, HP-6025 (IgG4; FITC; cat# F9890); e) from SouthernBiotech, HP6023 (mouse anti-human IgG_4_ pFc’; HRP; 1:20,000; cat# 9190–05), f) from NHP Reagent Resource, 1B3 (anti-rhesus IgG1/3; HRP; 1:5000).

The following unconjugated antibodies were used: a) PA-14 (Leronlimab; CytoDyn), conjugated in-house to PacBlu using Pacific Blue Antibody labeling Kit (ThermoFisher) and used at approximately 1:80 depending on the efficacy of conjugation, b) anti-idiotype antibody, PA-22 (CytoDyn). Live/dead Fixable Yellow Dead Cell Stain Kit and Near-IR Dead Cell Stain Kit (ThermoFisher) were amine-reactive dyes used at 1:1000 dilution to assess cell viability.

### Statistics

Two-way repeated measures analysis of variance (ANOVA) was used for longitudinal outcome measures to compare plasma viral load and normalized change from baseline for %CCR5, lymphocytes, CD4+, and CD8+ cells between the Leronlimab-treated and control groups over the 15-week study period. Two-way repeated measures ANOVA was also used to compare tissue vDNA and tissue vRNA changes between treatment groups over six tissues: PBMC, lymph nodes (axillary and mesenteric), spleen, colon, and cervix. Since in a typical experiment using repeated measures, two measurements taken at adjacent times are more highly correlated than two measurements taken several timepoints apart, optimal covariance structure chosen by Bayesian Information Criteria (BIC) was used to account for within-subject correlation. One-way ANOVA was used to compare peak and area-under-curve plasma viral load between treatment groups. Due to right-skewed data, some analyses were performed on log-transformed data. For normally distributed data, analyses were conducted on untransformed data, including normalized change from baseline for %CCR5 of CD8+ T-cells, lymphocytes, CD4+ T-cell, and CD8+ T-cell. Multiplicity adjusted p-values following Tukey procedures were presented. Statistical significance was determined at the significant alpha level of 0.05. Analyses were performed using SAS version 9.4, specifically PROC MIXED.

## Supporting information

S1 Table HIV-1 RNA copies/mL above LOD (>40 copies/mL) and the corresponding study week. List of all study week timepoints with plasma HIV-1 RNA copies/mL values higher than the limit of detection (>40 copies/mL).
(XLSX)

S2 Table Percent of total immune cells in long-term Leronlimab treatment in humans. Table shows immune cell percentages in the five HIV+ human participants from cohorts 1 and 2 and the median values of five healthy uninfected humans. P-values from unpaired t-test; pink highlighted boxes have p-values <0.05.
(DOC)

S3 Table Rhesus macaque demographics. Table shows animal ID, gender, age, and MHC alleles. N/A = not sequenced.
(XLSX)

S4 Table Individual P-values for longitudinal panels in macaques. Table shows the weekly P-values for longitudinal panels in plasma viral load (Fig 3D), CCR5+ CD4+ T-cells (Fig 3E), CCR5+ CD8+ T-cells (S7H Fig), lymphocyte (S5B Fig), CD4+ T-cell (S5C Fig), and CD8+ T-cell (S5D Fig). P-values were calculated by two-way repeated measures ANOVA with Tukey-Kramer adjustment. P-value <0.05 was significant and highlighted in orange.
(XLSX)

S5 Table Summary of tissue concentrations corresponding to Fig 4A and 4B. Ax LN = axillary lymph node, Ing LN = inguinal lymph node, and Mes LN = mesenteric lymph node. Tissue concentration represented as Leronlimab (ng) over total protein (mg)
(XLSX)

S6 Table 30-marker antibody human CyTOF Panel. 30 markers used in the human CyTOF panels grouped by antibody blone and metal tag.
(XLSX)

S1 Fig Serum chemistry of human participant. Serum values are shown for cohort 1 (n = 2; red) and cohort 2 (n = 3; blue). For each participant, longitudinal **(A)** individual timepoints and **(B)** mean (±SD) timepoints of alanine aminotransferase (ALT), alkaline phosphatase (ALP), aspartate aminotransferase (AST), total bilirubin, total protein, creatinine, and blood urea nitrogen (BUN). The two horizontal dotted lines in all panels represent the normal ranges in healthy, uninfected humans.
(TIF)

S2 Fig CyTOF immune profiling of participants. Cen-se (Cauchy-Enhanced Nearest-neighbor Stochastic Embedding) plots of immune cell type percentages of human participants receiving Leronlimab. A = 01–061; B = 01–064; C = 01–037; D = 01–057; E = 01–038.
(TIF)

S3 Fig CyTOF immune profiling between Leronlimab-treated HIV+ participants and untreated healthy participants. Showing plots for cell subsets that are statistically significantly different between Leronlimab-treated HIV+ participants and untreated healthy participants.
(TIF)

S4 Fig CCR5 receptor occupancy (RO) assays. **(A-B)** Representative flow cytometric analysis for (A) Eq 1 and (B) Eq 2 used to calculate for CCR5 RO by Leronlimab. **(C)** Representative flow cytometry plots displaying the different components required to calculate each equation, and at different levels of CCR5 RO: fully occupied, partially occupied, and unoccupied. Eq 1 used the frequency of IgG4+ events within the CD45+, singlet, live, CD3+, CD4+/CD8-, and CCR5+ population. Eq 2 used the frequency of IgG4+ and Leronlimab-PB+ events within the CD45+, singlet, live, CD3+, CD4+/CD8-, and CCR5+ population. Table at right shows the calculated percentages of CCR5 RO using the two equations. **(D)** Graphic comparison of longitudinal CCR5 RO on CD4+ T-cells in the blood calculated by each equation. Dashed vertical lines represents Leronlimab treatment.
(TIF)

S5 Fig Longitudinal complete blood counts in macaques. Longitudinal values are shown for the control (n = 5; black) and Leronlimab-treated (n = 4; green) macaques. **(A)** Absolute counts for peripheral blood counts of platelets, hemoglobin, red blood cells, white blood cells, neutrophils, monocytes, eosinophils, basophils, lymphocytes, CD4+ T-cells, and CD8+ T-cells. The two horizontal dotted lines indicate standard reference range for each parameter in macaque housed at ONPRC. **(B-D)** Longitudinal mean (±SEM) for (B) lymphocytes, (C) CD4+ T-cells, and (D) CD8+ T-cells, and weekly P-values can be found in S4 Table. Gray box represents period of Leronlimab treatment.
(TIF)

S6 Fig Longitudinal serum chemistry parameters in macaques. Albumin (ALB), alkaline phosphatase (ALP), alanine aminotransferase (ALT), aspartate aminotransferase (AST), gamma-glutamyltransferase (GGT), glucose (GLU), blood urea nitrogen (BUN), creatinine (CREA), cholesterol (CHOL), and triglyceride (TRIG). The two horizontal dotted lines indicate standard reference ranges for each parameter of rhesus macaques housed at ONPRC. Gray box represents period of Leronlimab treatment.
(TIF)

S7 Fig Additional data corresponding to Fig 3. **(A)** Longitudinal CCR5 RO levels by Leronlimab on blood CCR5+ CD14+ monocytes in Leronlimab-treated macaques. **(B-C)** CCR5 RO levels of axillary lymph node (AxLN) of CCR5+ (B) CD4+ T-cells and (C) CD14+ monocytes. **(D)** Longitudinal anti-Leronlimab rhesus IgG levels in plasma. **(E-F)** Mean (±SEM) plasma viral load comparison of the control (n = 5) and Leronlimab-treated macaques (n = 4) for (E) peak plasma viral loads and (F) area-under-the curves (AUC); P-values were calculated with one-way ANOVA test. **(G)** Mean (±SEM) longitudinal plasma viral load. **(H)** Longitudinal changes in CCR5+ CD8+ T-cell frequency in blood. Graphs show fold change from baseline (week-0). Weekly P-values found in S4 Table were calculated by two-way repeated measures ANOVA with Tukey-Kramer adjustment. Gray box represents period of Leronlimab treatment.
(TIF)

S8 Fig Assessment of Env V3 loop sequences from SHIV-infected macaques. Plasma vRNA from six timepoints were isolated and *env* was sequenced. Two Leronlimab-treated animals, 32617 and 35721, had undetectable viremia at week-15 and thus excluded at that timepoint. **(A)** Amino acid alignment of Env sequences. Consensus sequence for “SHIV SF 162P3” serves as the reference sequence. “Stock” is the sequence of the challenge stock virus used to infect all the animals at the start of the study. “BR24N”, “HXB2”, “Ams.32”, and “CA28NL” are sequences of CXCR4-ultilizing isolates related to SHIVSF162P3. Dots denote identical amino acids and the dashes indicate gaps compared to the reference sequence. Legend at right denotes the variant frequency. Variants <2% were excluded. Red “X” residue indicates more than one variant with a frequency of >2% at that position. **(B)** Change in variant frequency for two control animals, 32739 and 34094, at position 13 of the V3 loop (or position 306 in Env protein).
(TIF)

S9 Fig Additional representative flow cytometry plots corresponding to Fig 4D. Representative flow cytometry plots showing the co-staining of anti-CCR5 (clone 3A9) and Leronlimab (by anti-human IgG4, clone HP-6025) on CD4+ T-cells from Leronlimab-treated macaque, 32617.
(TIF)
