# Supplementary material for: Suppression of human and simian immunodeficiency virus replication with the CCR5-specific antibody Leronlimab in two species
Source: PLoS Pathog. 2022 Mar 31;18(3):e1010396. doi: 10.1371/journal.ppat.1010396 (PMC8970399; doi:10.1371/journal.ppat.1010396)
Supplement: S2 Table — Table shows immune cell percentages in the five HIV+ human participants from cohorts 1 and 2 and the median values of five healthy uninfected humans. P-values from unpaired t-test; pink highlighted boxes have p-values <0.05. (DOC) [file ppat.1010396.s002.doc]

|  | **01-061** | **01-064** | **01-037** | **01-057** | **01-038** | **HIV uninfected controls (n=5) median** | **P-value** |
| --- | --- | --- | --- | --- | --- | --- | --- |
| **CD3 T cells** | 44.91 | 40.47 | 60.23 | 43.76 | 24.84 | 61.76 | **0.0079** |
| **CD8 T cells Total** | 16.03 | 19.86 | 26.36 | 24.13 | 13.22 | 32.15 | 0.1777 |
| **CD8 T cells Naive** | 3.36 | 2.92 | 5.76 | 2.86 | 1.25 | 1.85 | 0.9111 |
| **CD8 T cells Central Memory** | 0.52 | 1.40 | 1.37 | 0.58 | 1.07 | 2.10 | **0.0374** |
| **CD8 T cells Effector Memory** | 2.42 | 5.62 | 2.32 | 10.62 | 4.61 | 4.70 | 0.4883 |
| **CD8 T cells Terminal Effector** | 9.73 | 9.92 | 16.90 | 10.08 | 6.28 | 15.42 | 0.3147 |
| **CD4 T cells Total** | 22.92 | 12.50 | 31.33 | 16.45 | 11.06 | 20.52 | 0.423 |
| **CD4 T cells Naive** | 7.57 | 1.38 | 5.62 | 4.44 | 0.36 | 9.80 | 0.214 |
| **CD4 T cells Central Memory** | 5.42 | 4.94 | 14.40 | 5.07 | 4.83 | 3.74 | 0.4429 |
| **CD4 T cells Effector Memory** | 2.54 | 2.09 | 2.22 | 2.75 | 1.89 | 4.68 | 0.1471 |
| **CD4 T cells Terminal Effector** | 7.38 | 4.09 | 9.09 | 4.19 | 3.98 | 4.29 | 0.6382 |
| **Treg** | 0.13 | 0.12 | 0.19 | 0.09 | 0.09 | 0.66 | **0.0123** |
| **Th1-like** | 0.42 | 0.28 | 0.70 | 0.16 | 0.13 | 0.95 | 0.124 |
| **Th2-like** | 3.60 | 3.50 | 3.80 | 1.95 | 2.38 | 4.6 | 0.327 |
| **Th17-like** | 1.03 | 1.64 | 1.52 | 0.68 | 0.64 | 1.06 | 0.8276 |
| **Gamma Delta T Cells** | 5.75 | 7.45 | 2.41 | 3.18 | 0.56 | 1.61 | 0.5265 |
| **MAIT & NKT CD4- T Cells** | 0.22 | 0.65 | 0.13 | 0.00 | 0.00 | 0.88 | 0.0676 |
| **B Cells** | 7.33 | 4.23 | 6.00 | 13.65 | 7.38 | 5.81 | 0.3766 |
| **B Cells Naive** | 6.10 | 3.31 | 4.00 | 11.11 | 6.61 | 5.45 | 0.4768 |
| **B Cells Memory** | 1.16 | 0.91 | 1.98 | 2.46 | 0.77 | 0.43 | 0.1868 |
| **B Cells Plasmablasts** | 0.06 | 0.01 | 0.02 | 0.07 | 0.01 | 0.02 | 0.2072 |
| **NK Cells** | 11.64 | 14.57 | 13.03 | 6.05 | 35.37 | 6.87 | 0.0905 |
| **Early NK** | 2.98 | 5.23 | 3.10 | 1.34 | 7.32 | 2.65 | 0.2245 |
| **Late NK** | 8.66 | 9.34 | 9.93 | 4.71 | 28.04 | 4.22 | 0.0779 |
| **Monocytes Total** | 21.75 | 29.66 | 14.23 | 26.70 | 20.38 | 13.47 | **0.0145** |
| **Monocytes Classical** | 18.82 | 26.25 | 11.82 | 24.10 | 17.57 | 11.43 | **0.0248** |
| **Monocytes Transitional** | 1.93 | 2.56 | 1.61 | 2.12 | 2.14 | 0.79 | **0.001** |
| **Monocytes Non-Classical** | 1.00 | 0.84 | 0.80 | 0.48 | 0.67 | 0.24 | **0.019** |
| **Dendritic Cells** | 2.22 | 1.16 | 1.26 | 1.44 | 1.02 | 0.74 | **0.0285** |
| **pDC** | 0.08 | 0.35 | 0.22 | 0.10 | 0.13 | 0.23 | 0.3034 |
| **mDC** | 2.14 | 0.81 | 1.04 | 1.34 | 0.89 | 0.54 | **0.028** |
