# Supplementary figures and images for: Suppression of human and simian immunodeficiency virus replication with the CCR5-specific antibody Leronlimab in two species
Source: PLoS Pathog. 2022 Mar 31;18(3):e1010396. doi: 10.1371/journal.ppat.1010396 (PMC8970399; doi:10.1371/journal.ppat.1010396)

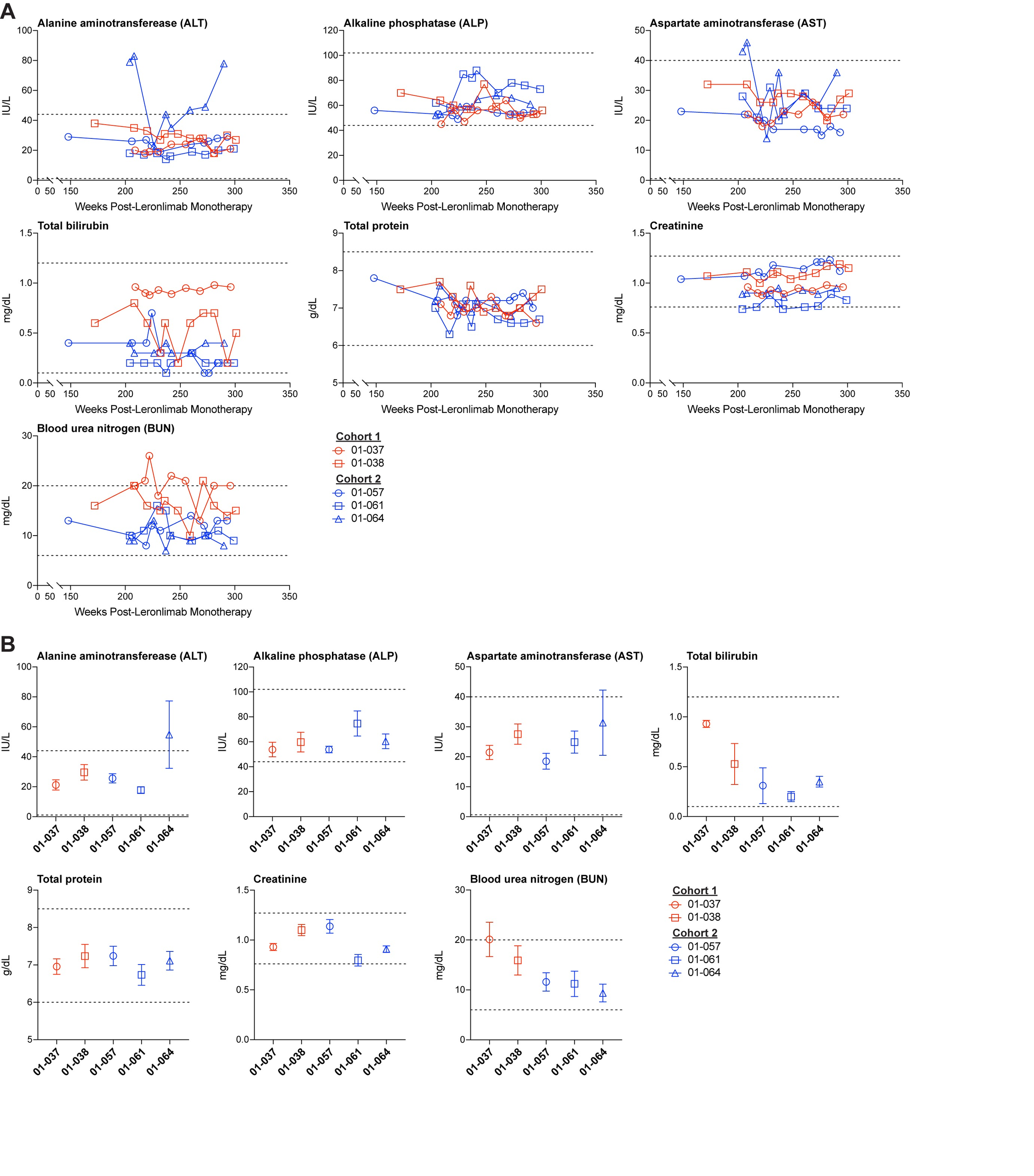

Supplement: S1 Fig — Serum values are shown for cohort 1 (n = 2; red) and cohort 2 (n = 3; blue). For each participant, longitudinal (A) individual timepoints and (B) mean (±SD) timepoints of alanine aminotransferase (ALT), alkaline phosphatase (ALP), aspartate aminotransferase (AST), total bilirubin, total protein, creatinine, and blood urea nitrogen (BUN). The two horizontal dotted lines in all panels represent the normal ranges in healthy, uninfected humans. (TIF) [file ppat.1010396.s007.tif]

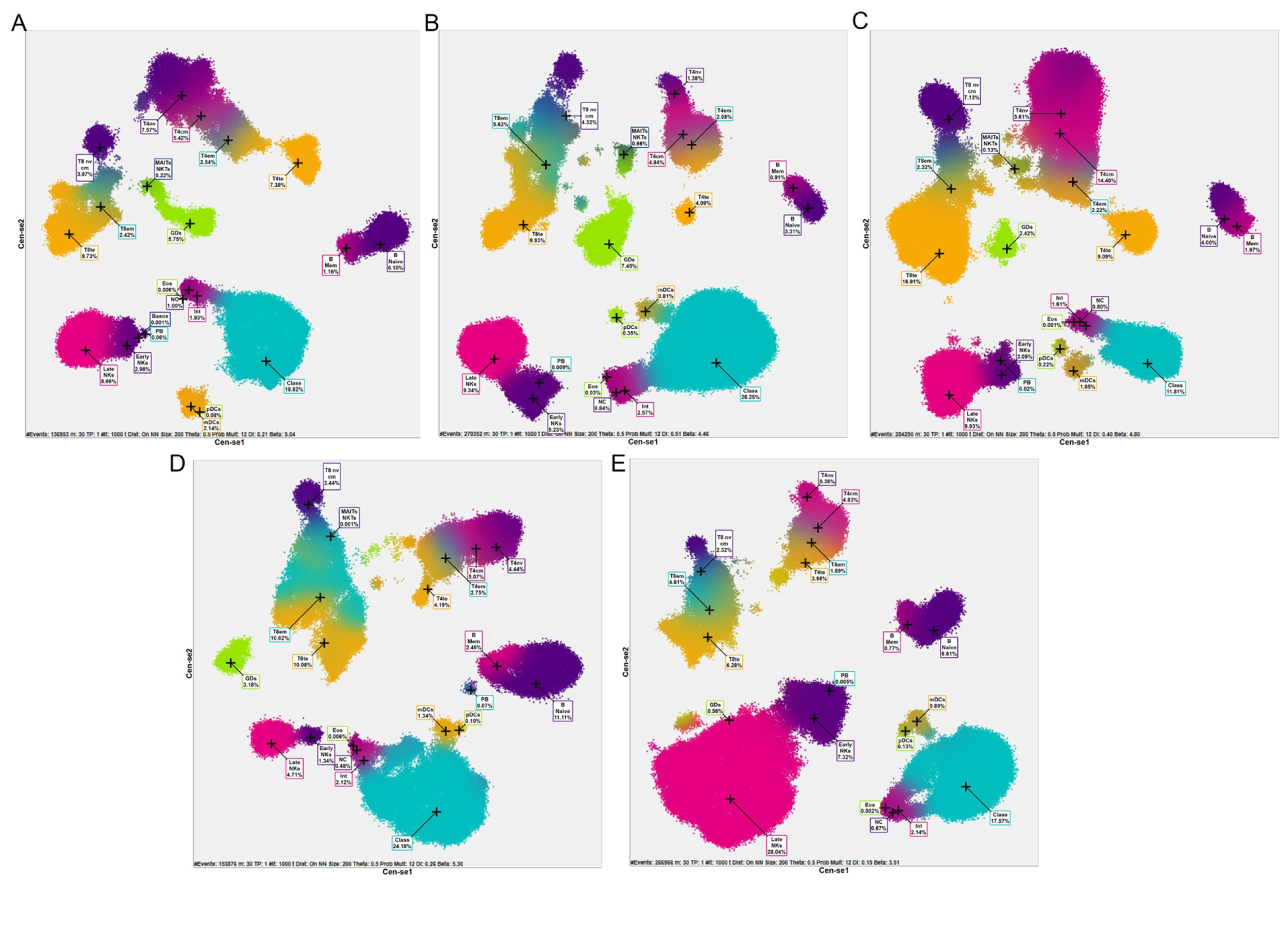

Supplement: S2 Fig — Cen-se (Cauchy-Enhanced Nearest-neighbor Stochastic Embedding) plots of immune cell type percentages of human participants receiving Leronlimab. A = 01–061; B = 01–064; C = 01–037; D = 01–057; E = 01–038. (TIF) [file ppat.1010396.s008.tif]

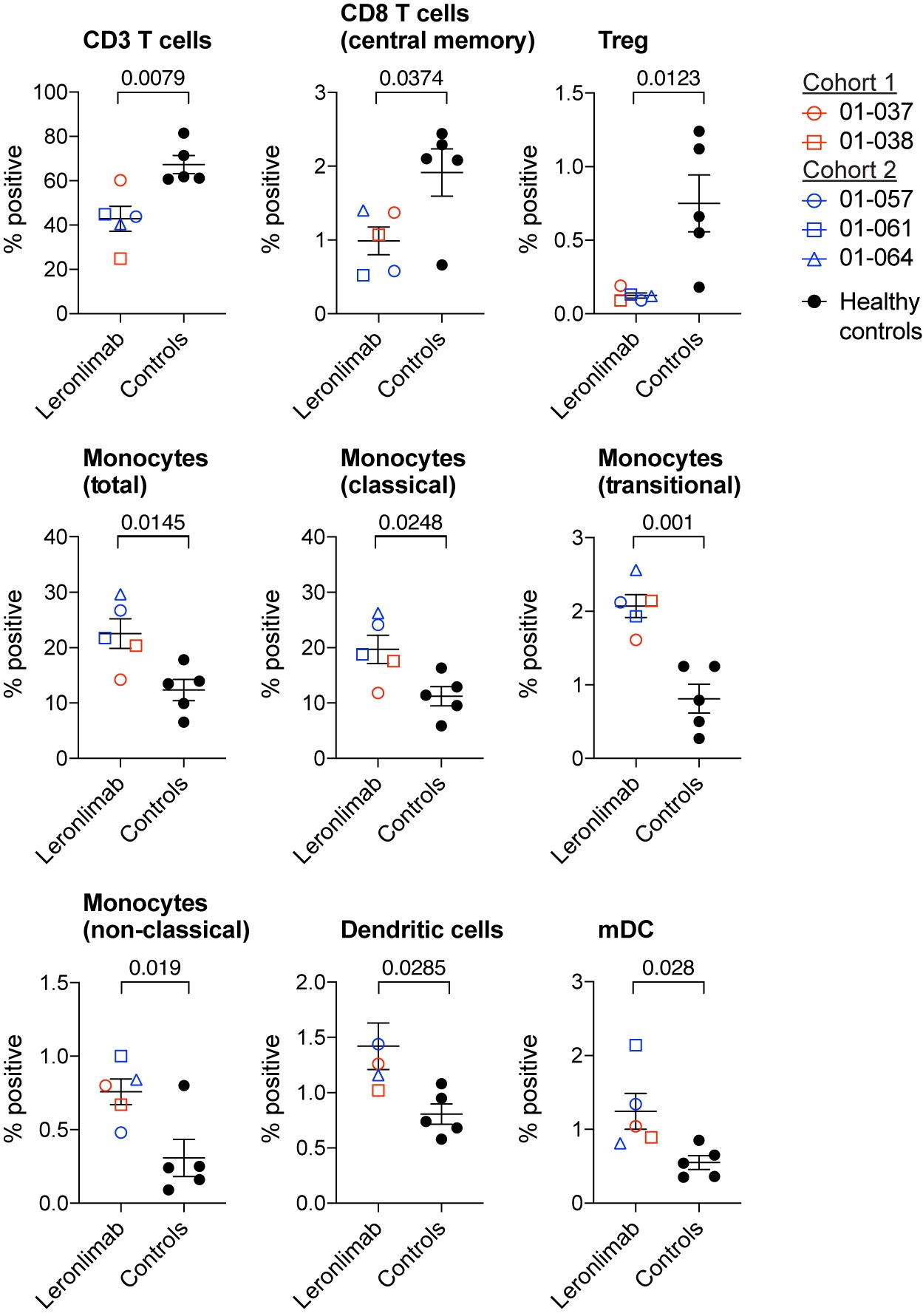

Supplement: S3 Fig — Showing plots for cell subsets that are statistically significantly different between Leronlimab-treated HIV+ participants and untreated healthy participants. (TIF) [file ppat.1010396.s009.tif]

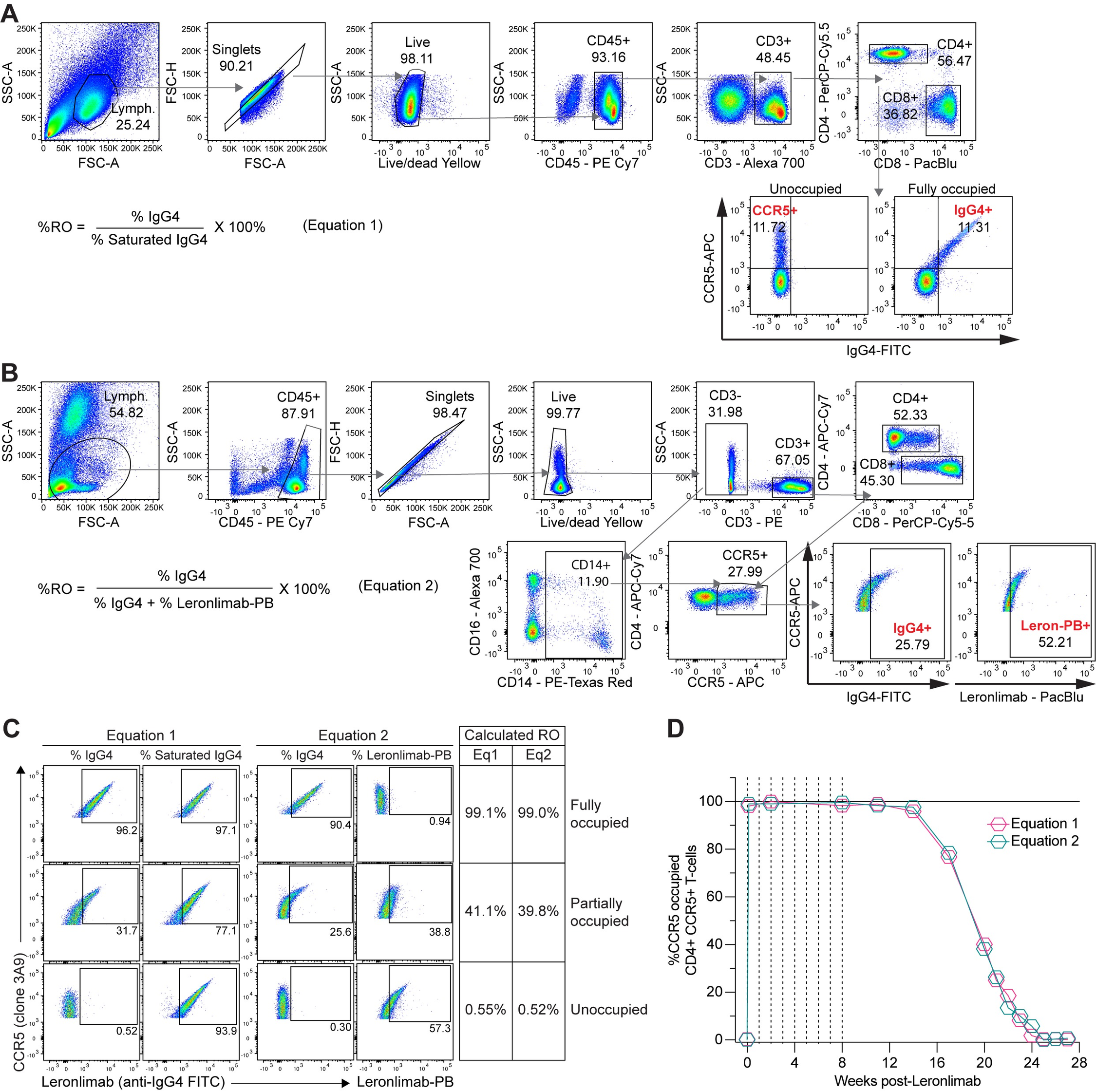

Supplement: S4 Fig — (A-B) Representative flow cytometric analysis for (A) Eq 1 and (B) Eq 2 used to calculate for CCR5 RO by Leronlimab. (C) Representative flow cytometry plots displaying the different components required to calculate each equation, and at different levels of CCR5 RO: fully occupied, partially occupied, and unoccupied. Eq 1 used the frequency of IgG4+ events within the CD45+, singlet, live, CD3+, CD4+/CD8-, and CCR5+ population. Eq 2 used the frequency of IgG4+ and Leronlimab-PB+ events within the CD45+, singlet, live, CD3+, CD4+/CD8-, and CCR5+ population. Table at right shows the calculated percentages of CCR5 RO using the two equations. (D) Graphic comparison of longitudinal CCR5 RO on CD4+ T-cells in the blood calculated by each equation. Dashed vertical lines represents Leronlimab treatment. (TIF) [file ppat.1010396.s010.tif]

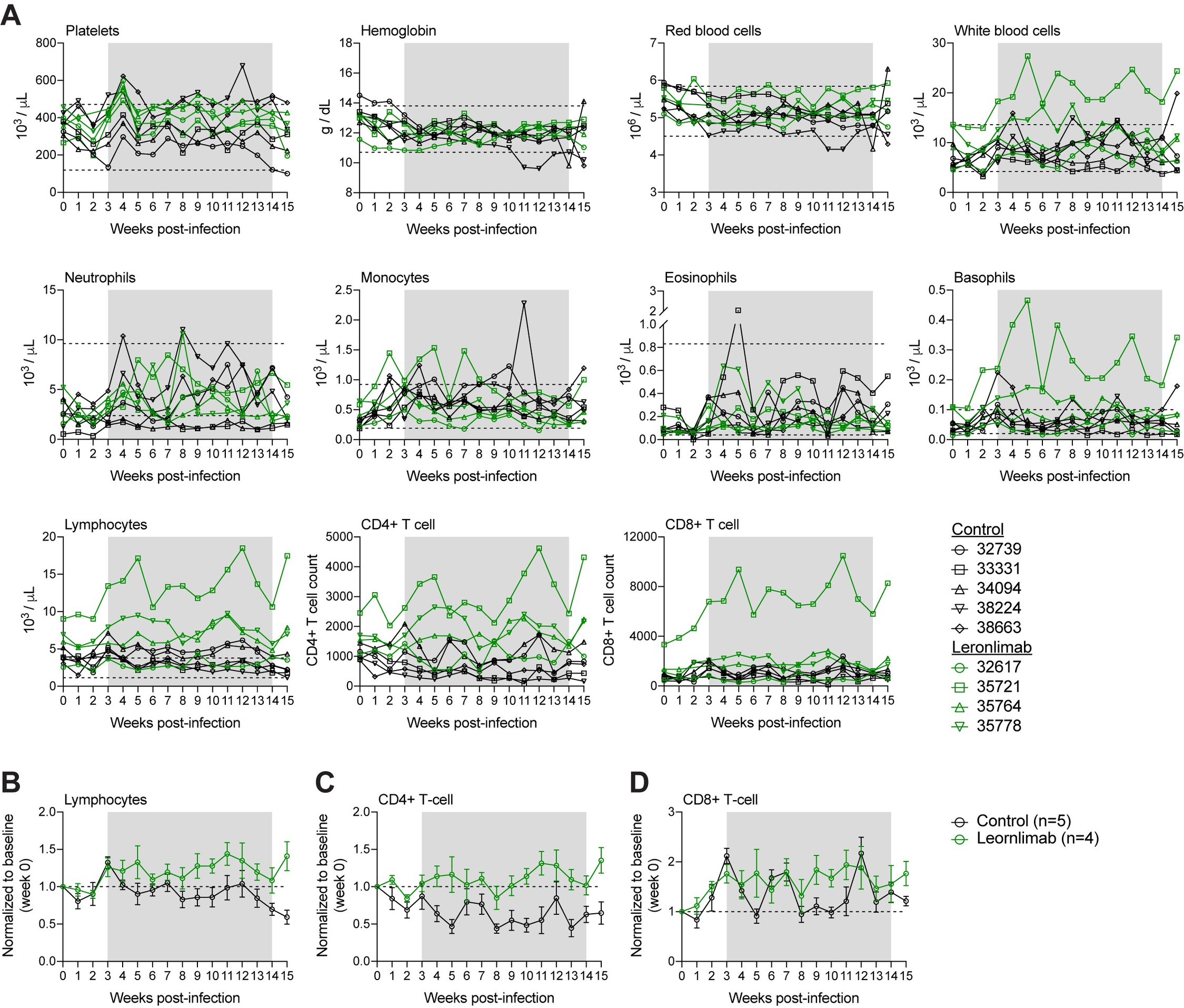

Supplement: S5 Fig — Longitudinal values are shown for the control (n = 5; black) and Leronlimab-treated (n = 4; green) macaques. (A) Absolute counts for peripheral blood counts of platelets, hemoglobin, red blood cells, white blood cells, neutrophils, monocytes, eosinophils, basophils, lymphocytes, CD4+ T-cells, and CD8+ T-cells. The two horizontal dotted lines indicate standard reference range for each parameter in macaque housed at ONPRC. (B-D) Longitudinal mean (±SEM) for (B) lymphocytes, (C) CD4+ T-cells, and (D) CD8+ T-cells, and weekly P-values can be found in S4 Table. Gray box represents period of Leronlimab treatment. (TIF) [file ppat.1010396.s011.tif]

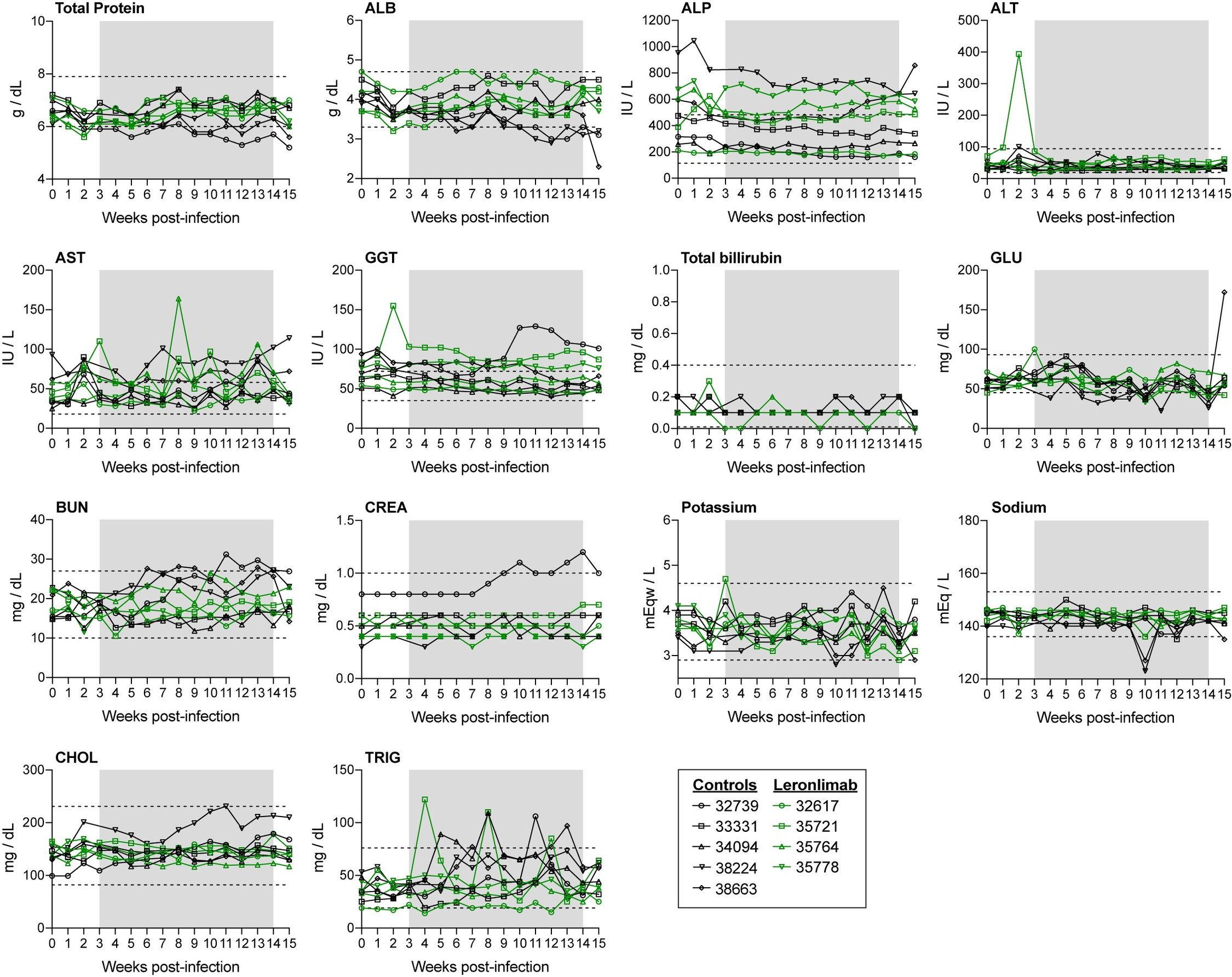

Supplement: S6 Fig — Albumin (ALB), alkaline phosphatase (ALP), alanine aminotransferase (ALT), aspartate aminotransferase (AST), gamma-glutamyltransferase (GGT), glucose (GLU), blood urea nitrogen (BUN), creatinine (CREA), cholesterol (CHOL), and triglyceride (TRIG). The two horizontal dotted lines indicate standard reference ranges for each parameter of rhesus macaques housed at ONPRC. Gray box represents period of Leronlimab treatment. (TIF) [file ppat.1010396.s012.tif]

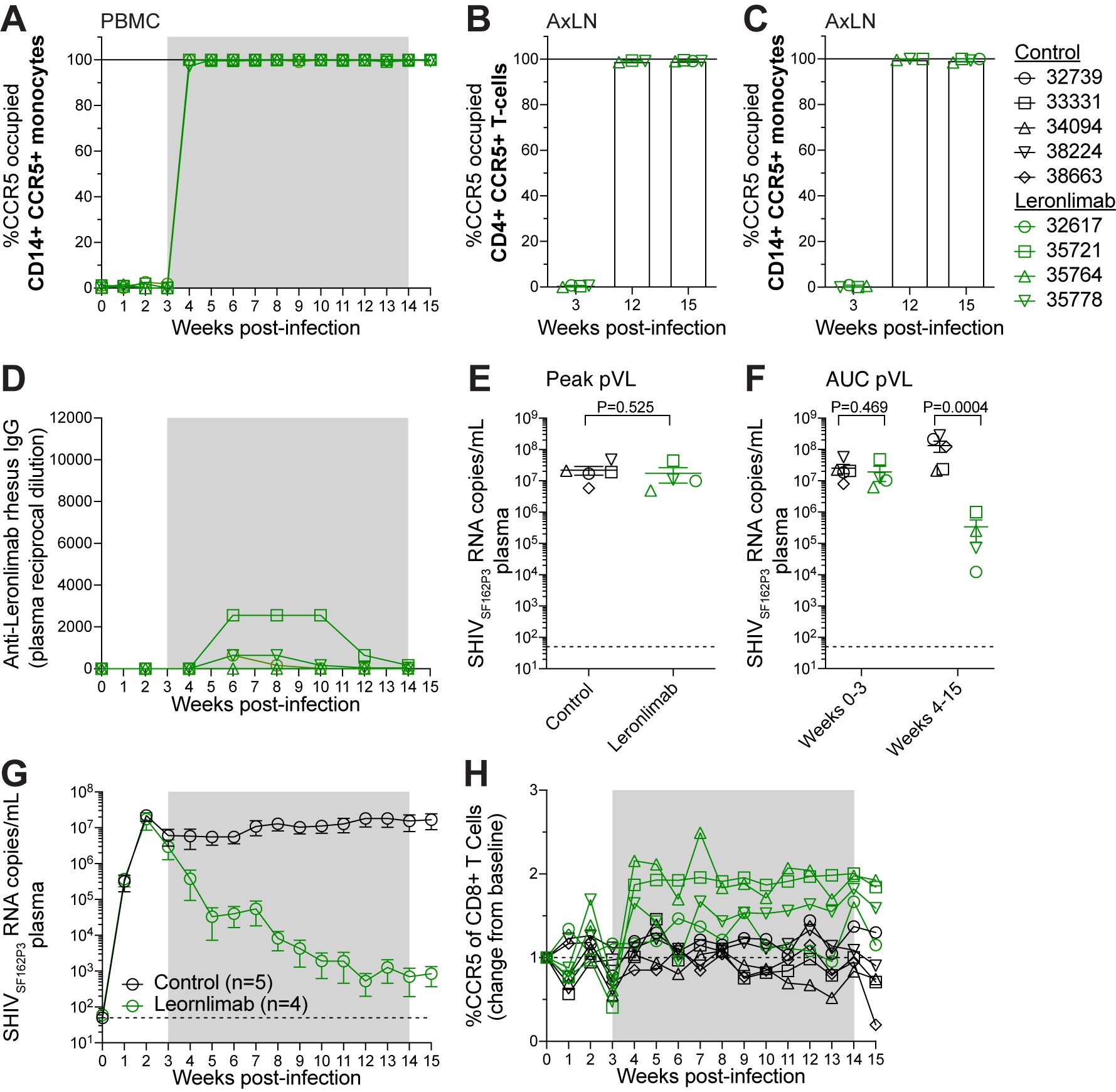

Supplement: S7 Fig — (A) Longitudinal CCR5 RO levels by Leronlimab on blood CCR5+ CD14+ monocytes in Leronlimab-treated macaques. (B-C) CCR5 RO levels of axillary lymph node (AxLN) of CCR5+ (B) CD4+ T-cells and (C) CD14+ monocytes. (D) Longitudinal anti-Leronlimab rhesus IgG levels in plasma. (E-F) Mean (±SEM) plasma viral load comparison of the control (n = 5) and Leronlimab-treated macaques (n = 4) for (E) peak plasma viral loads and (F) area-under-the curves (AUC); P-values were calculated with one-way ANOVA test. (G) Mean (±SEM) longitudinal plasma viral load. (H) Longitudinal changes in CCR5+ CD8+ T-cell frequency in blood. Graphs show fold change from baseline (week-0). Weekly P-values found in S4 Table were calculated by two-way repeated measures ANOVA with Tukey-Kramer adjustment. Gray box represents period of Leronlimab treatment. (TIF) [file ppat.1010396.s013.tif]

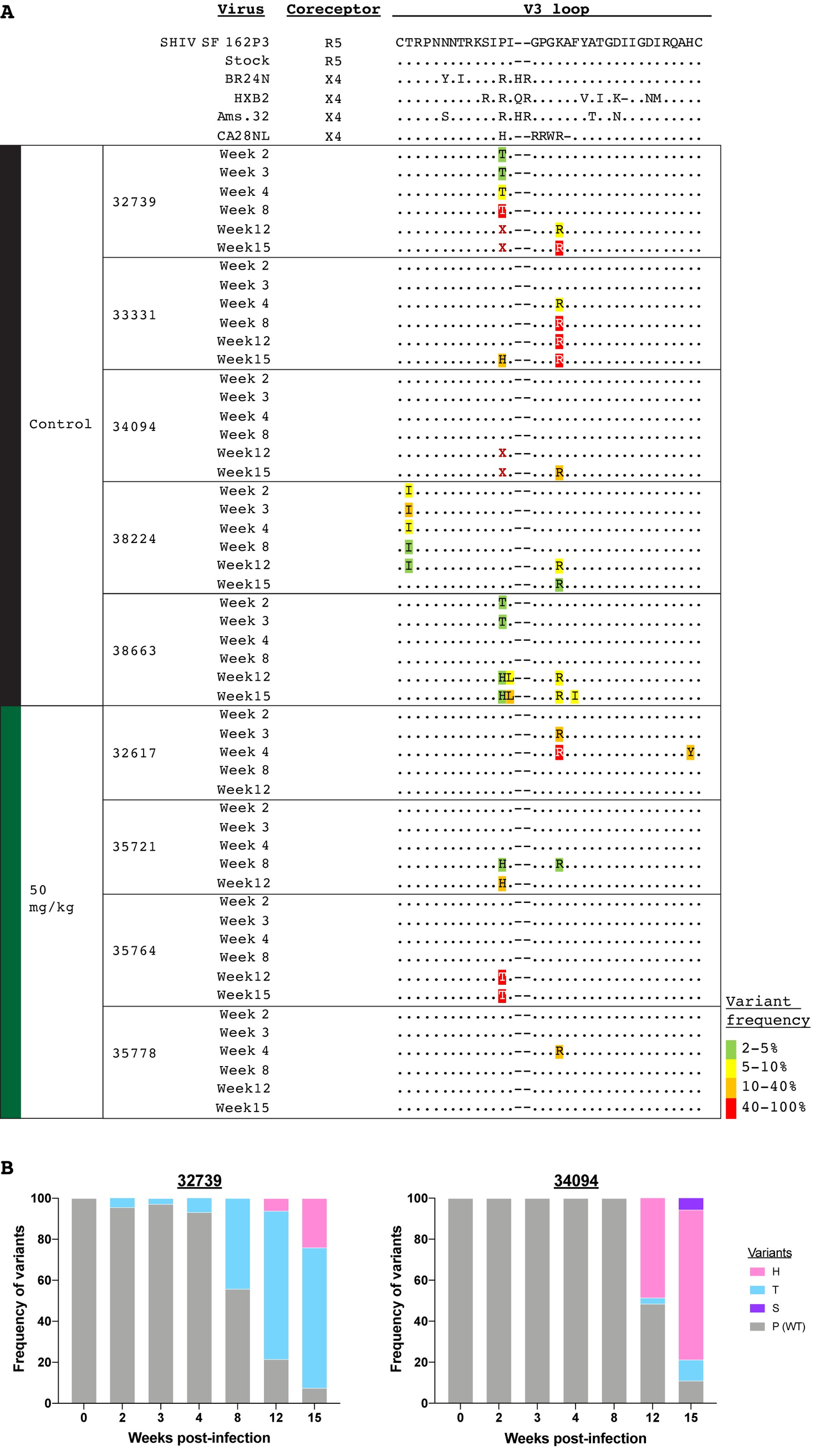

Supplement: S8 Fig — Plasma vRNA from six timepoints were isolated and env was sequenced. Two Leronlimab-treated animals, 32617 and 35721, had undetectable viremia at week-15 and thus excluded at that timepoint. (A) Amino acid alignment of Env sequences. Consensus sequence for “SHIV SF 162P3” serves as the reference sequence. “Stock” is the sequence of the challenge stock virus used to infect all the animals at the start of the study. “BR24N”, “HXB2”, “Ams.32”, and “CA28NL” are sequences of CXCR4-ultilizing isolates related to SHIVSF162P3. Dots denote identical amino acids and the dashes indicate gaps compared to the reference sequence. Legend at right denotes the variant frequency. Variants <2% were excluded. Red “X” residue indicates more than one variant with a frequency of >2% at that position. (B) Change in variant frequency for two control animals, 32739 and 34094, at position 13 of the V3 loop (or position 306 in Env protein). (TIF) [file ppat.1010396.s014.tif]

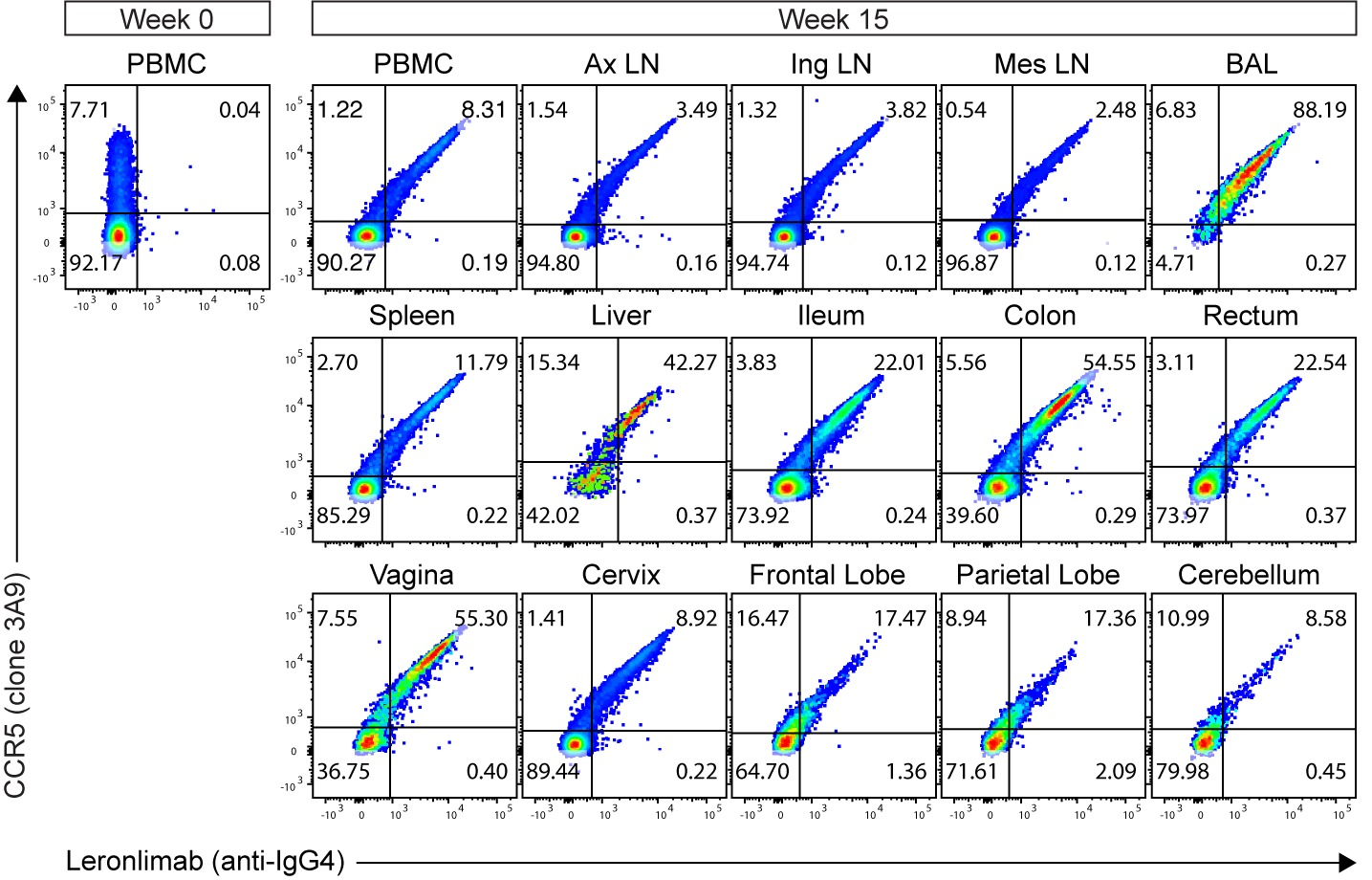

Supplement: S9 Fig — Representative flow cytometry plots showing the co-staining of anti-CCR5 (clone 3A9) and Leronlimab (by anti-human IgG4, clone HP-6025) on CD4+ T-cells from Leronlimab-treated macaque, 32617. (TIF) [file ppat.1010396.s015.tif]
